# High-Fidelity Cytosine Base Editing in a GC-Rich Corynebacterium glutamicum with Reduced DNA Off-Target Editing Effects

**DOI:** 10.1128/spectrum.03760-22

**Published:** 2022-11-14

**Authors:** Yu Been Heo, Gue-Ho Hwang, Seok Won Kang, Sangsu Bae, Han Min Woo

**Affiliations:** a Department of Food Science and Biotechnology, Sungkyunkwan University, Suwon, Republic of Korea; b BioFoundry Research Center, Institute of Biotechnology and Bioengineering, Sungkyunkwan University, Suwon, Republic of Korea; c Department of Chemistry and Research Institute for Convergence of Basic Sciences, Hanyang University, Seoul, Republic of Korea; d Department of Biochemistry and Molecular Biology, Seoul National University College of Medicine, Jongno-gu, Seoul, Republic of Korea; Ocean University of China

**Keywords:** genome editing, cytosine base editor, off-target, *Corynebacterium glutamicum*, nonsense mutation, CRISPR, industrial bacteria, gene editing

## Abstract

Genome editing technology is a powerful tool for programming microbial cell factories. However, rat APOBEC1-derived cytosine base editor (CBE) that converts C•G to T•A at target genes induced DNA off-targets, regardless of single-guide RNA (sgRNA) sequences. Although the high efficiencies of the bacterial CBEs have been developed, a risk of unidentified off-targets impeded genome editing for microbial cell factories. To address the issues, we demonstrate the genome engineering of Corynebacterium glutamicum as a GC-rich model industrial bacterium by generating premature termination codons (PTCs) in desired genes using high-fidelity cytosine base editors (CBEs). Through this CBE-STOP approach of introducing specific cytosine conversions, we constructed several single-gene-inactivated strains for three genes (*ldh*, *idsA*, and *pyc*) with high base editing efficiencies of average 95.6% (*n* = 45, C6 position) and the highest success rate of up to 100% for PTCs and ultimately developed a strain with five genes (*ldh*, *actA*, *ackA*, *pqo*, and *pta*) that were inactivated sequentially for enhancing succinate production. Although these mutant strains showed the desired phenotypes, whole-genome sequencing (WGS) data revealed that genome-wide point mutations occurred in each strain and further accumulated according to the duration of CBE plasmids. To lower the undesirable mutations, high-fidelity CBEs (pCoryne-YE1-BE3 and pCoryne-BE3-R132E) was employed for single or multiplexed genome editing in C. glutamicum, resulting in drastically reduced sgRNA-independent off-targets. Thus, we provide a CRISPR-assisted bacterial genome engineering tool with an average high efficiency of 90.5% (*n* = 76, C5 or C6 position) at the desired targets.

**IMPORTANCE** Rat APOBEC1-derived cytosine base editor (CBE) that converts C•G to T•A at target genes induced DNA off-targets, regardless of single-guide RNA (sgRNA) sequences. Although the high efficiencies of bacterial CBEs have been developed, a risk of unidentified off-targets impeded genome editing for microbial cell factories. To address the issues, we identified the DNA off-targets for single and multiple genome engineering of the industrial bacterium Corynebacterium glutamicum using whole-genome sequencing. Further, we developed the high-fidelity (HF)-CBE with significantly reduced off-targets with comparable efficiency and precision. We believe that our DNA off-target analysis and the HF-CBE can promote CRISPR-assisted genome engineering over conventional gene manipulation tools by providing a markerless genetic tool without need for a foreign DNA donor.

## INTRODUCTION

Engineering bacteria is in high demand not only for producing biomaterials or bioproducts (1–3) but also for medical use such as therapeutics and diagnostics (4). To do so, a precise targeted genome editing tool is crucial to obtain the desired mutations efficiently and rapidly. Previous targeted genome editing technologies have been dependent to single or double homologous recombination (HR) events. However, with single HRs using SacB (encoding levansucrase lethal to the microbial host) and a counterselection system (i.e., antibiotics-sensitive), it is difficult to select the correct genotype with low efficiency and double crossover events (5, 6). To promote genetic engineering of the HR, one-step gene inactivation in Escherichia coli requires the phase λ-Red recombinase and the linear DNAs that include a homologous region and a selection marker. After the target gene was disrupted using double HR, the selection marker can be removed by using the flippase recombination (7). Still, this procedure leaves behind a scar (82 to 85 nt), which is potentially problematic under multiplex engineering when a new PCR cassette can recombine at the existing scars. For markerless deletion, I-SecI endonuclease-mediated DNA double-strand breaks (DSBs) trigger DNA repair via HR, by which genome engineering facilitates in-frame deletion or small insertions/deletions within homologous arms. This also requires a two-step process of a selection and counterselection system in the targeted locus (8). To overcome the limitations of the HR, the advent of genetic engineering tools such as clustered regularly interspaced short palindromic repeat (CRISPR)-associated technologies have extensively contributed to this field, along with *in silico* design of protospacers, optimization of transformations, and cloning of CRISPR RNAs (crRNAs) (9, 10).

Although high frequencies of the desired mutants were obtained using CRISPR-associated HR technologies, foreign donor DNA templates that contain the homologous and edited regions must be accompanied to yield the desired mutations (11) via homology-directed repair. In addition, the CRISPR-Cas system kills nonedited bacterial cells, which caused occasional escapers to avoid DNA cleavages up on the toxicity of the Cas9 nuclease at elevated expression (9, 12, 13). Potential escapers limit the identification of the desired mutant with highly efficient genome engineering (9). Previously, we and other groups harnessed original CRISPR effectors, including CRISPR-Cas9, CRISPR-Cas12a, CRISPR interference (CRISPRi), and CRISPR activation (CRISPRa) for engineering various industrial bacteria species such as Corynebacterium glutamicum, E. coli, and Streptomyces coelicolor. Following those original CRISPR effectors, new genetic engineering tools such as DNA base editors (BEs) have been developed to promote genome engineering without foreign DNA insertions and with less toxic Cas nickase.

BEs, involving cytosine base editors (CBEs) and adenine base editors (ABEs), can induce point mutations at desired sites in the absence of donor DNA templates without generating DSBs (13, 14). In particular, CBE-mediated C•G-to-T•A conversion can introduce a premature termination codon (PTC) to the coding strand of a desired gene, causing early translational termination and resulting in gene inactivation (previously named CRISPR-STOP [15] or i-STOP [16]). Given these advantages, bacterial genome engineering using BEs has been expected to play a key role in building efficient microbial cell factories for producing value-added bioproducts. To date, a few studies have applied CBEs such as BE3 and Target-AID for genetic engineering in microbial cells (10, 17). The Kondo group used Target-AID (18) for base editing in E. coli to perform multiplex mutagenesis of transposases genes on 41 loci; the Weber and Lee groups used BE3 in S. coelicolor, named CRISPR-cBEST (19), to inactivate two-copies of the genes in the kirromycin biosynthetic pathway and to perform a multiplexed editing using a Csy4 endoribonuclease; and the Ma group demonstrated Target-AID (20) for genome engineering in C. glutamicum to enhance glutamate production using multiplex base editing and to generate a gene inactivation library for 94 transcriptional factors.

However, it was revealed that CBEs showed promiscuous cytosine editing in the genome (21, 22) and in transcripts (23) regardless of single-guide RNA (sgRNA) sequences, which were generated by a cytidine deaminase of CBE. Therefore, to reduce these sgRNA-independent off-target effects, cytidine deaminase should be further engineered. For this purpose, Yang and colleagues developed high-fidelity versions of CBEs (24), named YE1-BE3 and BE3-R132E, in which rAPOBEC1 of BE3 was engineered to contain double mutations (W90Y+R126E) or a single mutation (R132E), respectively. Thus, systematic features of CBEs, including genome-wide DNA off-target effects, have not seriously addressed thus far. A risk of unidentified off-targets could impede next-generation CRISPR-guided genome engineering for microbial cell factories in the community.

To address these issues, we developed rAPOBEC1-derived CBE-mediated base editing in C. glutamicum as a GC-rich model bacterium and constructed several single gene-inactivated strains for three genes (*ldh*, *idsA*, and *pyc*), as well as a strain with inactivation of five genes (*ldh*, *actA*, *ackA*, *pqo*, and *pta*), for enhancing succinate production as a model production system. For these strains, we profiled genome-wide DNA off-target sites via whole-genome sequencing (WGS) and found tens of single-nucleotide variants (SNVs) in genome. We further revealed that most off-target mutations were derived by nonspecific DNA deaminase activity of CBEs and that the off-target editing effects were significantly reduced by high-fidelity versions of CBEs (i.e., YE1-BE3 and R132E-BE3) in either single or multiplexed genome editing (MGE). DNA off-target analysis and the high-fidelity (HF)-CBE can promote reliable CRISPR-assisted genome engineering over conventional gene manipulation tools by providing a markerless genetic tool with high editing efficiency and by reducing undesired mutations.

## RESULTS

### Schematic of CBE-STOP and *in silico* investigation of applicable targets for CBE-STOP in C. glutamicum.

**FIG 1:**
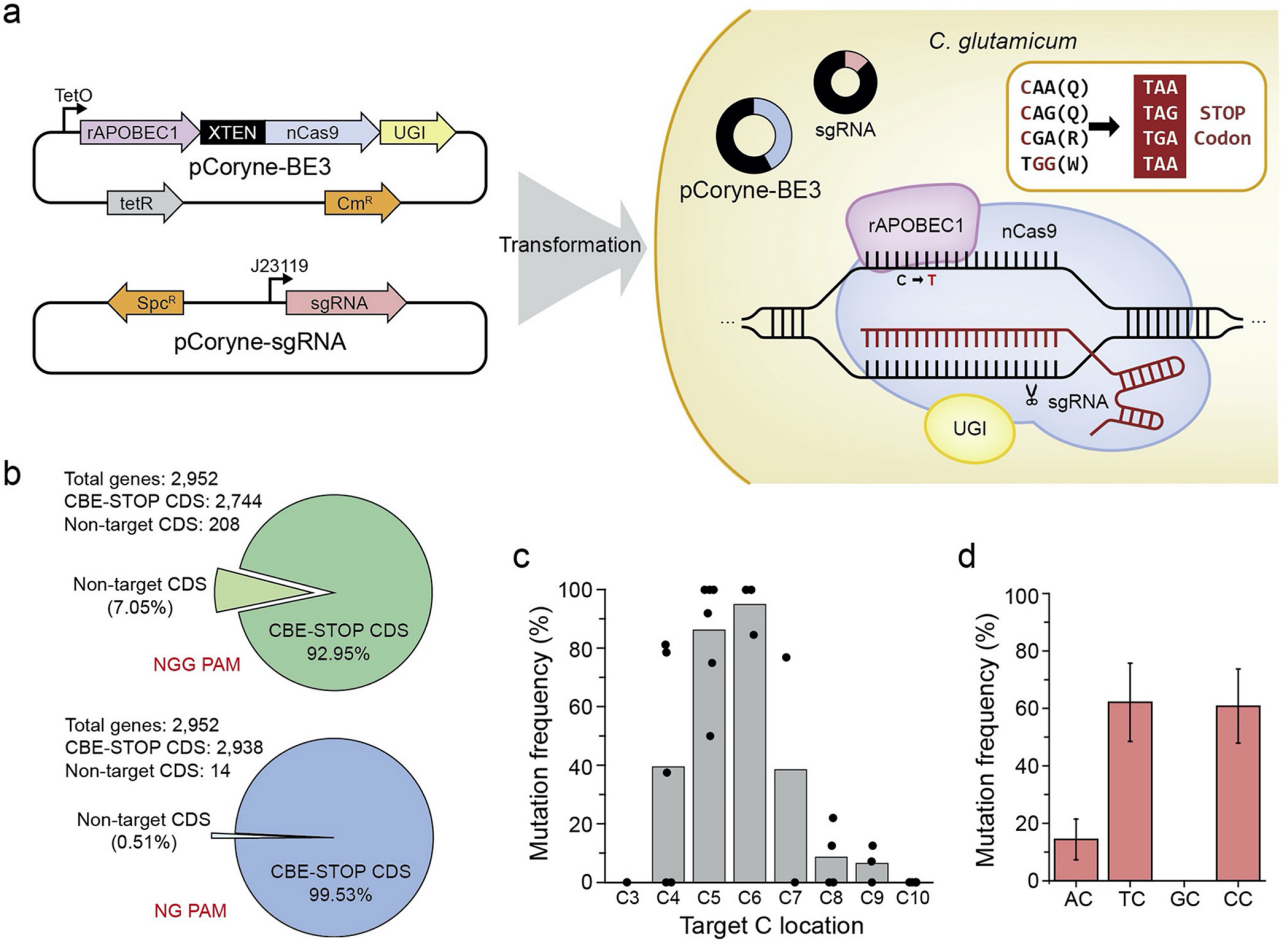
Construction of cytosine base editor (CBE)-STOP for genetically engineering C. glutamicum and the features of pCoryne-BE3. (a) Scheme of CBE using pCoryne-BE3 and pCoryne2-single-guide RNA (sgRNA) in C. glutamicum. After DNA replication or repair, a C•G-to-T•A conversion occurred. CBE-STOP generates stop codons (TGA, TAG, or TAA) in the coding strand sequence by converting the targeted C to T of CAA (Gln), CAG (Gln), or CGA (Arg) in the coding strand and in the noncoding strand of TGG (Trp) at the editing window. The protospacer adjacent motif (PAM) site for the CBE-STOP was 5′-NGG. (b) Targetable coding sequences by CBE-STOP with at least one stop codon in the C4 to C8 position (Fig. S1) using either 5′-NGG PAM or 5′-NG PAM. (c) Mutation frequencies (%) or base editing performance (%) at the editing-window positions. Points in bars represents each mutation frequency for eight different protospacers at editable window (C3 to C12). See the detail data (Table S6). (d) Mutation frequencies (%) for sequence-context dependency. C4 positions were only available with the GC motif. The error bar represents the standard deviation (SD). CDS, coding sequence; UGI, uracil glycosylase inhibitor; *C. glutamicum, Coryebacterium glutamicum*; rAPOBEC1, rat cytidine deaminase; XTEN, a 16 amino acid flexible linker; nCas9, a DNA nickase; sgRNA, single guide RNA; Spc, spectinomycin; Cm, chloramphenicol.

As described above, CBEs can introduce a PTC to the coding strand of a desired gene, which ultimately results in gene inactivation. For example, three different codons, 5′-CAA-3′ (Gln), 5′-CAG-3′ (Gln), and 5′-CAA-3′ (Arg), in the coding strand can turn to STOP codons of 5′-TAA-3′, 5′-TAG-3′, and 5′-TGA-3′, respectively, while 3′-ACC-5′ (Trp) on the noncoding strand (i.e., 5′-TGG-3′ in the coding strand) can turn to a STOP codon of 3′-ATT-5′ (i.e., 5′-TAA-3′) (Fig. 1a).

To facilitate use of CBEs for generating stop codons (CBE-STOP, here) in C. glutamicum, we first inspected all targetable sites in the C. glutamicum genome (ATCC 13032; NC_003450) *in silico* to investigate how many coding genes can be potentially disrupted by the CBE-STOP approach. The results showed that of a total of 2,952 genes, 92.95% were targetable by a conventional CBE that recognizes a canonical protospacer adjacent motif (PAM), 5′-NGG-3′ (Fig. 1b; Fig. S1), whereas most genes (99.53%) were covered by an expanded version, NG-CBE, that recognizes a noncanonical PAM, 5′-NG-3′ (Table S1; Data Set S1) (25), suggesting the versatile utility of CBE-STOP in C. glutamicum.

### Editing features of pCoryne-BE3, cytosine base editor.

Next, we constructed strain-specific CBE versions by adopting two CBE variants (BE1 and BE3) developed by the Liu group (14) that were named pCoryne-BE1 [rAPOBEC1-XTEN-dCas9(D10A/H840A)] and pCoryne-BE3 [rAPOBEC1-XTEN-nCas9(D10A)-UGI], respectively. We chose an sgRNA, named ldh-sg134, which could target cytosine at the codon for amino acid 134 (Trp) in the *ldh* gene, encoding a lactate dehydrogenase. To compare the editing ability of pCoryne-BE1 and pCoryne-BE3 constructs, ldh-sg134 was cotransferred with either pCoryne-BE1 or pCoryne-BE3 into C. glutamicum (Tables S2 and S3). From the spread bulk populations, we arbitrarily picked 10 colonies in each pCoryne-BE1- and pCoryne-BE3-transferred group and performed Sanger sequencing for all. The results showed that pCoryne-BE3 (100%) exhibited better editing efficiency than pCoryne-BE1 (80%) (Fig. S2), consistent with previous results in human cells (14). Therefore, we decided to use pCoryne-BE3 for the CBE-STOP approach in further experiments.

To examine the position-dependent editing activity of pCoryne-BE3, eight different targets, including ldh-sg134, were selected (Table S3) in *idsA* (geranylgeranyl pyrophosphate synthase), *pyc* (pyruvate carboxylase), and *ldh* genes. After the transformation of each sgRNA with pCoryne-BE3 in C. glutamicum, a total of 103 independent colonies were arbitrarily picked and subjected to Sanger sequencing. The results revealed that the pCoryne-BE3 showed the best editing performance for cytosine at the C5 (86.3%, *n* = 73 colonies) and C6 (95.6%, *n* = 45 colonies) positions (Fig. 1c; Table S4). Notably, further analysis of sequences adjacent to the target cytosine indicated that pCoryne-BE3 has a strong preference for the TC motif, similar to previous results in human cells as T**C** > C**C** > A**C** > G**C** (Fig. 1d; Table S5).

### Single gene knockout for *ldh*, *pyc*, and *idsA* via CBE-STOP.

**FIG 2:**
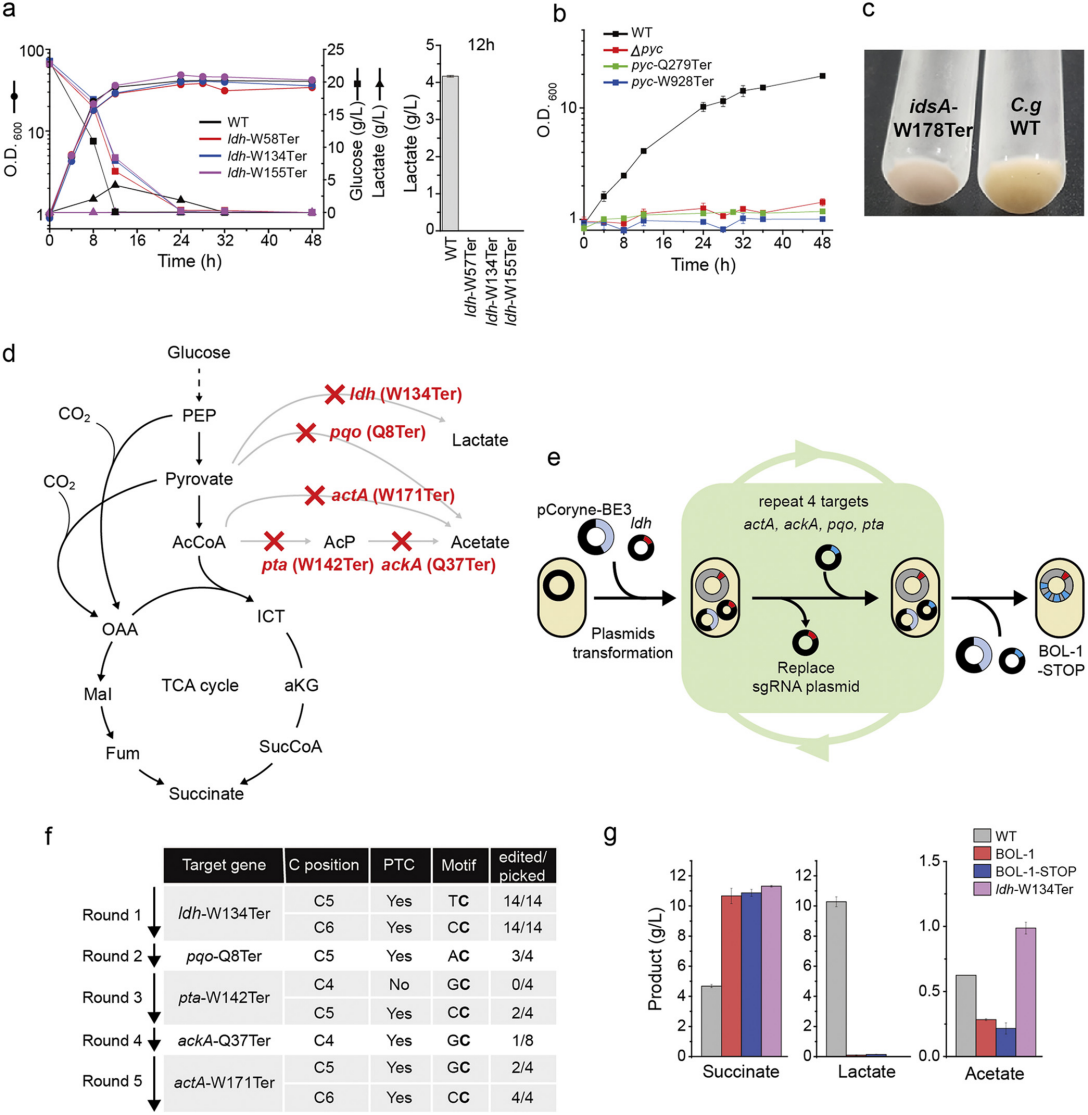
Application of CBE-STOP for single or multiple gene inactivation in C. glutamicum. (a) Phenotypic results of WT and *ldh*-STOP mutants (*ldh*-W57Ter, *ldh*-W134Ter, and *ldh*-R155Ter). Each *ldh* mutant with a different genotype constructed with CBE-STOP showed comparable growth rate and glucose consumption capacity compared to WT (left). Solid circles, optical density at 600 nm (O.D._600_); squares, glucose; black, WT; red, *ldh*-W57Ter; blue, *ldh*-W134Ter; purple, *ldh*-R155Ter. Cultivations were done in at least triplicate. The data represent mean values, and the error bars represent the standard deviations. *ldh*-STOP mutant showed no lactate product 12 h after transformation (right). (b) Phenotypic results of WT (black), Δ*pyc* (red), *pyc*-Q279Ter (green), and *pyc*-W928Ter (blue). (c) Phenotypic results of the cell pellets of wild type (WT) (right) and *idsA*-W178Ter (left). (d) A reconstruction of the metabolic pathway for succinate production in BOL-1-STOP. (e) Flow diagram for a single CBE-STOP and multiple CBE-STOPs. (f) Mutation frequencies of the multiplexed CBE-STOP mutants to construct BOL-1-STOP in a sequential order (*ldh* → *pqo* → *pta* → *ackA* → *actA*). (g) Production of organic acids (succinate, lactate, adn acetate) by WT, BOL-1, BOL-1-STOP, and *ldh*-W134Ter. The error bars represent the standard deviation (SD).

Based on the features of pCoryne-BE3 in C. glutamicum, we investigated whether CBE-STOP could be applied for precise microbial engineering to reconstruct metabolic pathways or optimize microbial hosts. To this end, three different Ldh-deficient STOP strains, named *ldh*-W57Ter, *ldh*-W134Ter, and *ldh*-R155Ter, were obtained using pCoryne-BE3 with different sgRNAs and cultivated under aerobic conditions. For all three strains, we confirmed that no lactate was produced in the aerobic culture (Fig. 2a), suggesting that CBE-STOP could be a flexible tool for target positions. In parallel, we also applied CBE-STOP to engineer *idsA* and *pyc* genes in the same way, using sgRNAs targeting the corresponding genes. As a result, we observed no cell growth of Pyc-deficient STOP strains (*pyc*-Q279Ter and *pyc*-W928Ter) under conditions where lactate was the carbon source (Fig. 2b) and white cell pellets of the IdsA-deficient STOP strain (*idsA*-W178Ter) (Fig. 2c), consistent with previous studies with in-frame deleted strains (26, 27). For all STOP mutant strains, we confirmed the genotypes using Sanger sequencing (Fig. S3). Taken together, we concluded that CBE-STOP can be a practical method for microbial genome engineering.

### Five genes knockout step by step using CBE-STOP.

Next, we sought to sequentially apply CBE-STOP for multiple gene inactivation as MGE to construct microbial cell factories. As a model system, we decided to create a BOL-1-STOP strain to produce succinate, a C_4_-dicarboxylic acid, as a monomer for sustainable chemical industry, in C. glutamicum. Although strain development requires excessive metabolic engineering, C. glutamicum BOL-1 (in-frame Δ*cat*, Δ*pqo*, Δ*pta-ack*, Δ*ldhA*) (Table 1; Fig. 2d) has been used as a basic succinate producer under anaerobic conditions (27). We started with the *ldh*-W134Ter strain constructed above. After curing the sgRNA plasmids (ldh-sg134), we transferred sgRNA targeting the *pqo* gene and selected colonies containing the intended mutations. We repeated these MGE experiments for *pta*, *ackA*, and *actA* genes in tandem (Fig. 2e; Fig. S4 and S5). According to the preference of the target motif and the C position, the base editing efficiencies were varied in CBE-STOP genome engineering (Fig. 2f). Inactivation for *pqo* showed high frequencies of 75% (3 of 4) at the C5 position. Also, inactivation for *pta* and *actA*, of which motifs were CC, exhibited with high frequencies of 50% (2 of 4; C5 position) and 100% (4 of 4; C6 position and a bystander), respectively. However, the editing efficiency of *ackA* with the GC motif (1 of 8; C5 position) was substantially lower than those of the other targets above. None of the protospacers with other motifs was available to *ackA*. As a result, the final editing efficiency for the five target genes depends on a single target with low efficiency as the rate-limiting step (in this case, the *ackA* gene) because each round has an independent procedure for base editing with intrinsic motif preferences and editable window position. Ultimately, we obtained a BOL-1-STOP strain in which a total of five genes were inactivated as introducing PTC using the serial MGE. For the comparison of the succinate production, the BOL-1-STOP strain produced 10.8 g/liter succinate from 2% (wt/vol) glucose as the sole carbon, which was comparable to the previous results with the BOL-1 strain (Fig. 2g). In addition, the BOL-1-STOP strain showed obviously reduced acetate production compared to the *ldh*-W134Ter strain, similar to the original BOL-1 strain.

**TABLE 1:** Bacterial strains and plasmids used^*a*^

Strain or plasmid	Relevant characteristics	Reference
Strains		
E. coli DH5α	F-(80d *lac*Z M15) (*lac*ZYA-*arg*F) U169 *hsd*R17(r^–^ m^+^) *rec*A1 *end*A1 *rel*A1 *deo*R96	40
C. glutamicum WT	ATCC 13032, wild-type strain	ATCC
C. glutamicum *ldh*-W57Ter	C. glutamicum WT derivative with nonsense mutations of *ldh*-W57Ter using CBE-STOP	This study
C. glutamicum *ldh*-W134Ter	C. glutamicum WT derivative with nonsense mutations of *ldh*-W134Ter using CBE-STOP	This study
C. glutamicum *ldh*-R155Ter	C. glutamicum WT derivative with nonsense mutations of *ldh*-R155Ter using CBE-STOP	This study
C. glutamicum *ldh*-W134Ter_R132E	C. glutamicum WT derivative with nonsense mutations of *ldh*-W134Ter using HF-CBE-STOP (BE3-R132E)	This study
C. glutamicum *ldh*-W134Ter_YE1	C. glutamicum WT derivative with nonsense mutations of *ldh*-W134Ter using HF-CBE-STOP (YE1-BE3)	This study
C. glutamicum *idsA*-W69Ter	C. glutamicum WT derivative with nonsense mutations of *idsA-*W69Ter using CBE-STOP	This study
C. glutamicum *idsA-*W178Ter	C. glutamicum WT derivative with nonsense mutations of *idsA-*W178Ter using CBE-STOP	This study
C. glutamicum *idsA*-Q337Ter	C. glutamicum WT derivative with nonsense mutations of *idsA-*Q337Ter using CBE-STOP	This study
C. glutamicum *pyc*-Q279Ter	C. glutamicum WT derivative with nonsense mutations of *pyc-*Q279Ter using CBE-STOP	This study
C. glutamicum *pyc*-W928Ter	C. glutamicum WT derivative with nonsense mutations of *pyc-W*928Ter using CBE-STOP	This study
C. glutamicum BOL-1	C. glutamicum WT derivative with in-frame deletions of *actA(cat)*, *pqo*, *pta-ack*, and *ldhA*	27
C. glutamicum BOL-1-STOP	C. glutamicum WT derivative with nonsense mutations of *actA-*W171Ter, *pqo-*Q8Ter, *pta-*W142Ter*, ack-*Q37Ter, and *ldh-*W134Ter	This study
C. glutamicum *ldh*-*pqo*-*pta*	C. glutamicum WT derivative with nonsense mutations of *ldh*-W134Ter, *pqo*-Q8Ter, *pta*-Q96Ter	This study
C. glutamicum *sdhCD*-*sdhA*-*sdhB*	C. glutamicum WT derivative with nonsense mutations of *sdhCD*-Q73Ter, *sdhA*-Q294Ter-*sdhB*-Q10Ter	This study
C. glutamicum *sdhCD*-*sdhA*-*sdhB*-*pta*	C. glutamicum WT derivative with nonsense mutations of *sdhCD*-Q73Ter, *sdhA*-Q294Ter-*sdhB*-Q10Ter, *pta*-Q96Ter	This study
Plasmids		
pBbEB2c-RFP	ColE1 (*Ec*), pBL1 (*Cg*), Cm^r^, P*_tetA_*, BglBrick sites, *rfp*, CoryneBrick vector	42, 45
pET28b-BE1	BE1; His6-rAPOBEC1-XTEN-dCas9, Km^r^, Addgene 73018	14
pET42b-BE3	BE3; GGS-His6-rAPOBEC-XTEN-nCas9-GGS-UGI-GGS-NLS, Km^r^, [nCas9; Cas9(D10A)], Addgene 87437	46
pCoryne-BE1	pBbEB2c-derived vector carrying the rat APOBEC1-XTEN-dCas9 protein, Cm^r^	This study
pCoryne-BE3	pBbEB2c-derived vector carrying the rat APOBEC1-XTEN-nCas9-UGI protein, Cm^r^	This study
pCoryne-YE1-BE3	pCoryne-BE3-derived vector carrying the engineered YE1 [APOBEC1(W90Y, R126E)]	This study
pCoryne-BE3-R132E	pCoryne-BE3-derived vector carrying the engineered R132E [APOBEC1(R132E)]	This study
pJYS2_crtYF	Rep oriV (*Cg*) pMB1 oriV (*Ec*) Spc^r^, Pj23119-crRNA targeting *crtYf*	12
pgRNA-bacteria	ColE1 (*Ec*), Amp^r^, customizable guide RNA (gRNA), Addgene 44251	43
pCoryne2-sgRNA	pJYS2 derivative containing the Pj23119-sgRNA gene, EcoRI, BamHI, SpeI restriction enzyme sites, single-guide RNA targeting vector, Spc^r^	44
pCoryne2-ldh-sg57	pCoryne2-sgRNA carrying sgRNA-ldh-W57Ter, Spc^r^	This study
pCoryne2-ldh-sg134	pCoryne2-sgRNA carrying sgRNA-ldh-W134Ter, Spc^r^	This study
pCoryne2-ldh-sg155	pCoryne2-sgRNA carrying sgRNA-ldh-R155Ter, Spc^r^	This study
pCoryne2-idsA-sg69	pCoryne2-sgRNA carrying sgRNA-idsA-W69Ter, Spc^r^	This study
pCoryne2-idsA-sg178	pCoryne2-sgRNA carrying sgRNA-idsA-W178Ter, Spc^r^	This study
pCoryne2-idsA-sg337	pCoryne2-sgRNA carrying sgRNA-idsA-Q337Ter, Spc^r^	This study
pCoryne2-pyc-sg279	pCoryne2-sgRNA carrying sgRNA-pyc-Q279Ter, Spc^r^	This study
pCoryne2-pyc-sg928	pCoryne2-sgRNA carrying sgRNA-pyc-W928Ter, Spc^r^	This study
pCoryne2-pta-sg70	pCoryne2-sgRNA carrying sgRNA-pta-Q70Ter, Spc^r^	This study
pCoryne2-pta-sg96	pCoryne2-sgRNA carrying sgRNA-pta-Q96Ter, Spc^r^	
pCoryne2-ackA-sg37	pCoryne2-sgRNA carrying sgRNA-ackA-Q37Ter, Spc^r^	This study
pCoryne2-pqo-sg8	pCoryne2-sgRNA carrying sgRNA-pqo-Q8Ter, Spc^r^	This study
pCoryne2-actA-sg22	pCoryne2-sgRNA carrying sgRNA-actA-W171Ter, Spc^r^	This study
pCoryne2-actA-sg119	pCoryne2-sgRNA carrying sgRNA-actA-W171Ter, Spc^r^	This study
pCoryne2-actA-sg171	pCoryne2-sgRNA carrying sgRNA-actA-W171Ter, Spc^r^	This study
pCoryne2-ackA-sg37	pCoryne2-sgRNA carrying sgRNA-ackA-Q37Ter, Spc^r^	This study
pCoryne2-ackA-sg220	pCoryne2-sgRNA carrying sgRNA-ackA-Q220Ter, Spc^r^	This study
pCoryne2-ackA-sg308	pCoryne2-sgRNA carrying sgRNA-ackA-Q308Ter, Spc^r^	This study
pCoryne2-sdhCD-sg73	pCoryne2-sgRNA carrying sgRNA-sdhCD-Q73Ter, Spc^r^	This study
pCoryne2-sdhA-sg294	pCoryne2-sgRNA carrying sgRNA-sdhA-Q294Ter, Spc^r^	This study
pCoryne2-sdhB-sg10	pCoryne2-sgRNA carrying sgRNA-sdhB-Q10Ter, Spc^r^	This study
pCoryne2-AarI	pCoryne2-sgRNA derivative containing the AarI, EcoRI restriction enzyme sites for cloning multiple sgRNA targeting vector, Spc^r^	This study
pCoryne2-sdhCD-sdhA-sdhB	pCoryne2-sgRNA carrying sgRNA-sdhCD-Q73Ter-sdhA-Q294Ter-sdhB-Q10Ter, Spc^r^	This study
pCoryne2-pqo-pta-ackA	pCoryne2-sgRNA carrying sgRNA- pqo-Q8Ter-pta-Q96Ter-ackAQ308Ter, Spc^r^	This study

a CBE, cytosine base editor; crRNA, CRISPR RNA; HF, high-fidelity; sgRNA, single-guide RNA; WT, wild type; CDS, coding sequence; UGI, uracil glycosylase inhibitor; *C. glutamicum, Coryebacterium glutamicum*; rAPOBEC1, rat cytidine deaminase; XTEN, a 16 amino acid flexible linker; nCas9, a DNA nickase; Spc, spectinomycin; Cm, chloramphenicol.

### Profiling of genome-wide DNA mutations in *ldh* strain and BOL-1 strain using WGS.

**FIG 3:**
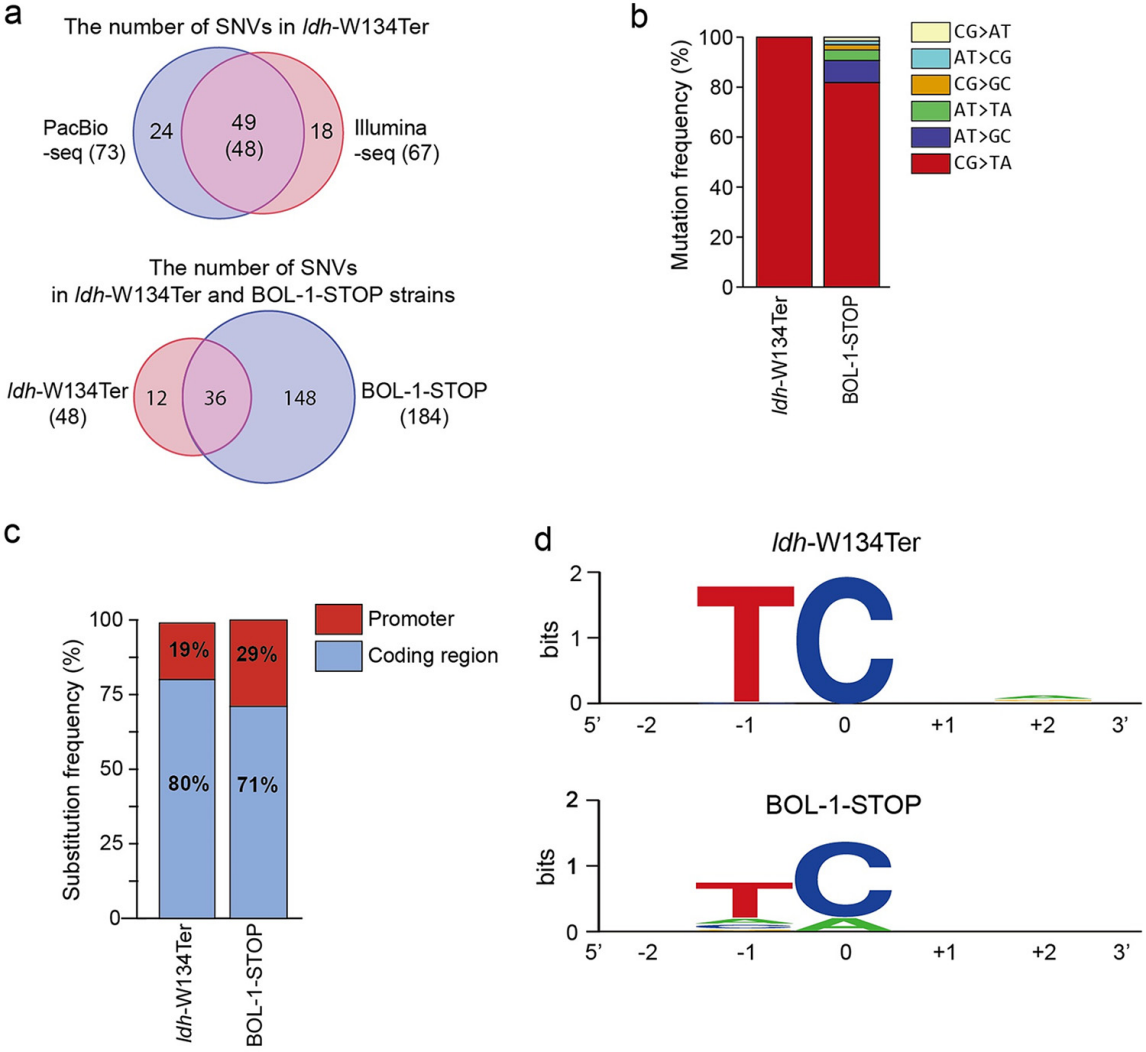
Genome-wide WGS analysis of the off-targets induced by CBE. (a) The number of substitutions in *ldh*-W134Ter mutant sequenced by either PacBio or Illumina. The number of substitutions confirmed by deep sequencing for *ldh*-W134Ter mutant and BOL-1-STOP. Whole-genome sequencing (WGS) was duplicated. (b) Mutation frequencies (%) of the bases for *ldh*-W134Ter mutant and BOL-1-STOP (c) Substitution frequencies (%) in either coding sequences or promoter regions in *ldh*-W134Ter mutant and BOL-1-STOP. (d) DNA logo for sequence context of the off-targets for either *ldh*-W134Ter mutant or BOL-1-STOP. The flanking sequences (2 bp on either side) were aligned at target cytosine positions. SNV, single-nucleotide variant.

Although the function of the BOL-1-STOP strain was in good agreement with the BOL-1 strain, it is necessary to reveal the genome-wide off-target effects in BOL-1-STOP. To this end, we performed whole-genome sequencing (WGS) for *ldh*-W134Ter and BOL-1-STOP strains, as well as wild-type C. glutamicum as a negative control. We first conducted both short-read sequencing using an Illumina HiSeq (Illumina-seq) and long-read sequencing using a PacBio platform (PacBio-seq) for the *ldh*-W134Ter strain. From the WGS outcomes, we obtained all SNVs information by aligning the data to a reference genome sequence (NC_003450) registered in the National Center for Biotechnology Information (NCBI) database and further filtered out mutations by removing substitutions commonly observed in the negative-control sample. As a result, we identified 73 SNVs from PacBio-seq and 67 SNVs from Illumina-seq, among which 49 SNVs excluding targeted bases (i.e., CC to TT in *ldh*-W134Ter) were observed in common (Fig. 3a; Table S6). We further confirmed that the 48 SNVs indeed existed in the *ldh*-W134Ter strain, although one SNV was a false positive, by conducting deep sequencing for each target. Only one nonsense (NCgl0352 encoding a hypothetical membrane protein), 8 missense, and 29 silent mutations were identified among 38 coding sequences (CDSs) in 48 SNVs (Table S6).

Using a similar procedure to Illumina-seq, we identified 184 SNVs (2 nonsense, 31 missense, and 97 silent mutations among 128 CDSs) from the BOL-1-STOP strain (Fig. 3a; Table S6) and compared them with 48 SNVs identified in the *ldh*-W134Ter strain. We initially expected that most SNVs in the *ldh*-W134Ter strain would be observed in the BOL-1-STOP strain, because we constructed the BOL-1-STOP strain sequentially based on the *ldh*-W134Ter strain. However, we found that 36 of 48 SNVs in the *ldh*-W134Ter strain were included in the BOL-1-STOP strain, but 12 SNVs were not. We assumed that the 12 SNVs were generated later in the process of removing the sgRNA plasmids, since pCoryne-BE3 could generate cytosine mutations in the absence of sgRNA. Likewise, we supposed that the remaining 148 SNVs in the BOL-1-STOP strain accumulated during tandem repeats of the CBE-STOP for four additional genes (*pqo*, *pta*, *ackA*, and *actA*), inducing approximately 4.5 times more mutations in BOL-1-STOP than *ldh*-W134Ter. Through SNV pattern analysis, we revealed that C•G-to-T•A substitutions were dominant in both *ldh*-W134Ter (98%) and BOL-1-STOP (82%) strains (Fig. 3b), strongly suggesting that most SNVs were produced by the cytosine deamination activity of pCoryne-BE3.

Next, we investigated where all SNVs in both strains were changed in the genomic loci. Based on the previous transcriptomic studies and the promoter information (28), we found that the SNVs generated in the strain *ldh*-W134Ter (48 SNVs) showed in the promoter region (19%) and coding region (80)% (Fig. 3c). After constructing the five gene knockout mutant (BOL-1-STOP) from the strain *ldh*-W134Ter, more SNVs were accumulated in the promoter region relative to the coding region, resulting in the promoter region (29%) and coding region (71%) (see the list of the SNVs in Table S6). Due to the recent report that the cytosine deaminase of BE3 (i.e., rAPOBEC1) effectively acts on single-stranded DNAs (ssDNAs) rather than double-stranded DNAs (dsDNAs) (29), we assumed that the off-target mutations might preferably occur near the promoter region when the repetitive genome editing is required. Further analysis of the sequence contexts neighboring each SNV revealed that 48 and 184 SNVs identified in *ldh*-W134Ter and BOL-1-STOP strains, respectively, exhibited a strong preference for the target motif, 5′-T**C** (Fig. 3d), also suggesting that these SNVs were directly derived from the promiscuous cytosine deamination activity of the pCoryne-BE3, consistent with previous results in human cells (30) and E. coli (31).

### Reduced off-target effects using high-fidelity CBEs (YE1 and R132E).

**FIG 4:**
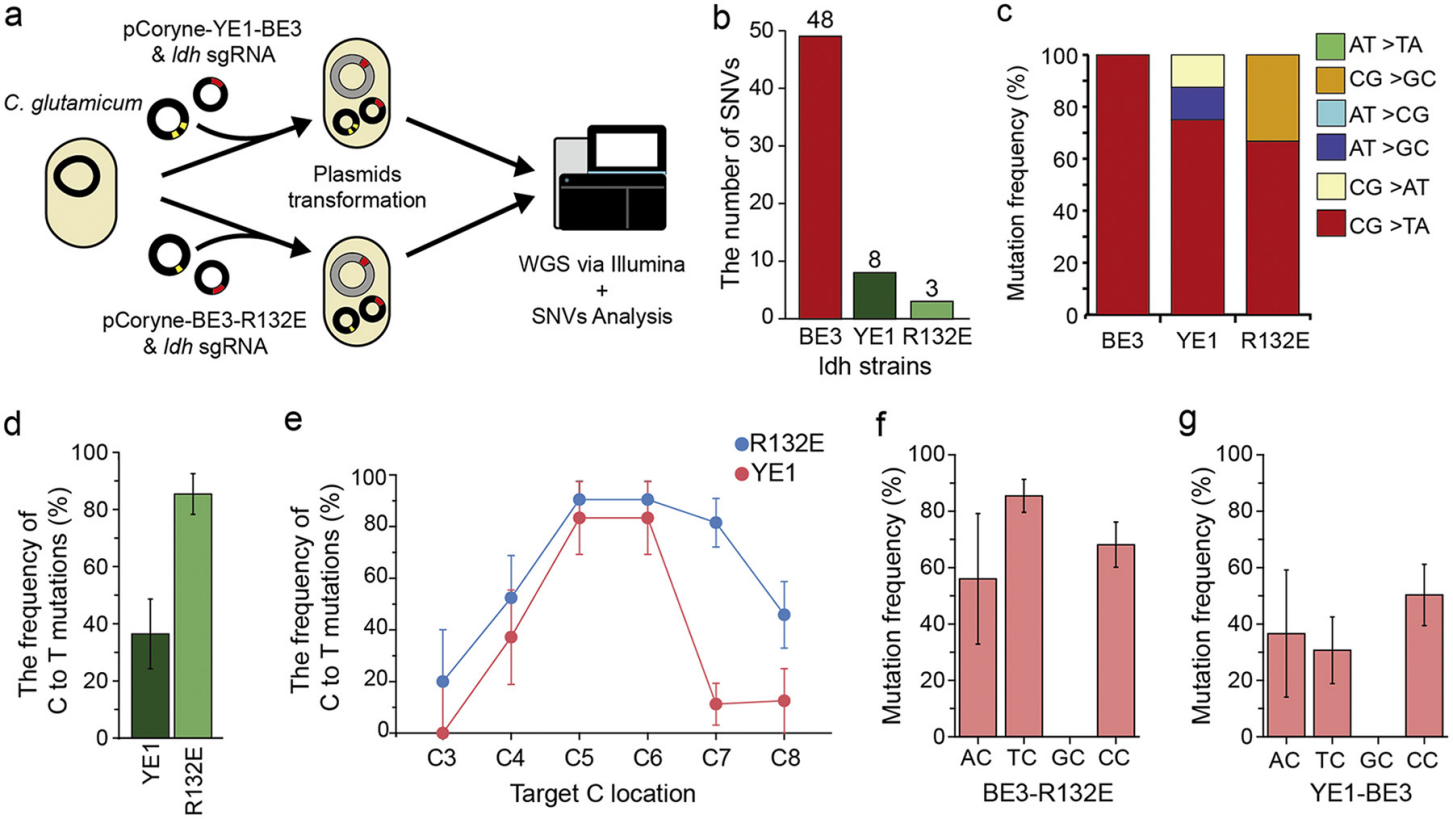
Genome-wide WGS analysis of the off-targets induced by high-fidelity (HF) CBE. (a) A scheme of the HF-CBE-STOP strain development of *ldh*-W134Ter using either YE1-BE3 or BE3-R132E. The WGS experiments were duplicated using Illumina. (b) Number of substitutions in *ldh*-W134Ter mutant using HF-CBEs, compared to original BE3. (c) Mutation frequencies (%) of the bases for *ldh*-W134Ter mutant using either BE3, YE1-BE3, or BE3-R132E. (d) Frequency of C-to-T mutations (%) for all sgRNAs tested using either YE1-BE3 (*n* = 94, colony), or BE3-R132E (*n* = 94, colony) (Table S8). (e) Mutation frequencies (%) or base editing performance (%) in arbitrary mutants at the editing-window positions using HF-CBEs. See the detail data (Table S8). (f) Mutation frequencies (%) for sequence-context dependency using HF-CBE (pCoryne-BE3-R132E). C4 and C7 positions were only available with the GC motif. The error bars represent the SD. (g) Mutation frequencies (%) for sequence-context dependency using HF-CBE (pCoryne-YE1-BE3) (Table S9). The C4 and C7 positions were only available with the GC motif.

To reduce these genome-wide DNA off-target effects, we further adopted the mutations in high-fidelity (HF) CBEs (24), YE1-BE3 and BE3-R132E, into the pCoryne-BE3, thereby constructing two HF-CBEs, named pCoryne-YE1-BE3 and pCoryne-BE3-R132E, respectively (Table 1). Next, we transferred each HF-CBE construct with ldh-sg134 into C. glutamicum and performed WGS using Illumina-seq for the selected strains (Fig. 4a). Notably, WGS data showed that the total number of SNVs was substantially reduced in both the pCoryne-YE1-BE3 (8 SNVs) and pCoryne-BE3-R132E (3 SNVs) transferred strains (Fig. 4b; Table S7), compared to the above data in the original pCoryne-BE3 transferred strain (48 SNVs), consistent with previous results in human cells (24). As when analyzing the off-target SNVs generated by either YE1-BE3 or BE3-R132E, the frequency of C to T mutations were 75% and 66%, respectively (Fig. 4c). These high C-to-T mutational frequencies suggested that the SNVs were generated by HF-CBEs as the off-target effects and were less likely obtained by spontaneous mutations.

To further examine the editing activities of both HF-CBEs, we tested each construct at an additional 16 endogenous target sites (Table S8), in which multiple cytosines were included at different positions (C3 to C8). Sanger sequencing results from edited strains revealed that pCoryne-BE3-R132E (85.4%, *n* = 94; colony) showed better editing efficiencies than pCoryne-YE1-BE3 (36.4%, *n* = 94; colony) (Fig. 4d; Table S8).

Further analysis of position-dependent editing activity revealed that both pCoryne-YE1-BE3 and pCoryne-BE3-R132E exhibited the best performance at the C5 and C6 positions, but pCoryne-YE1-BE3 had a relatively narrower editing window (C4 to C7), compared to pCoryne-BE3-R132E (C3 to C8), consistent with a previous study in human cells (32) (Fig. 4e). In case of the narrower C positions, pCoryne-BE3-R132E (90.5%, *n* = 76 colonies, C5 or C6 position) also showed better editing efficiencies than pCoryne-YE1-BE3 (82.5%, *n* = 66 colonies; C5 or C6 position) (Table S8). In addition, pCoryne-BE3-R132E possessed a sequence preference with a TC motif similar to that of pCoryne-BE3 (Fig. 4f and 4g), but pCoryne-YE1-BE3 showed slightly less preference. Lactate production was not observed in either HF-CBE-STOP strain (Fig. S6), suggesting the utility of HF-CBE-STOPs in C. glutamicum. Taken together, these results indicate that both HF-CBEs can be used as a more precise base editing strategy than the original pCoryne-BE3. Compared with pCoryne-YE1-BE3, pCoryne-BE3-R132E was chosen for further studies in C. glutamicum in terms of efficiency.

### Serial or simultaneous multiplexed genome editing using HF-CBE-STOP.

**FIG 5:**
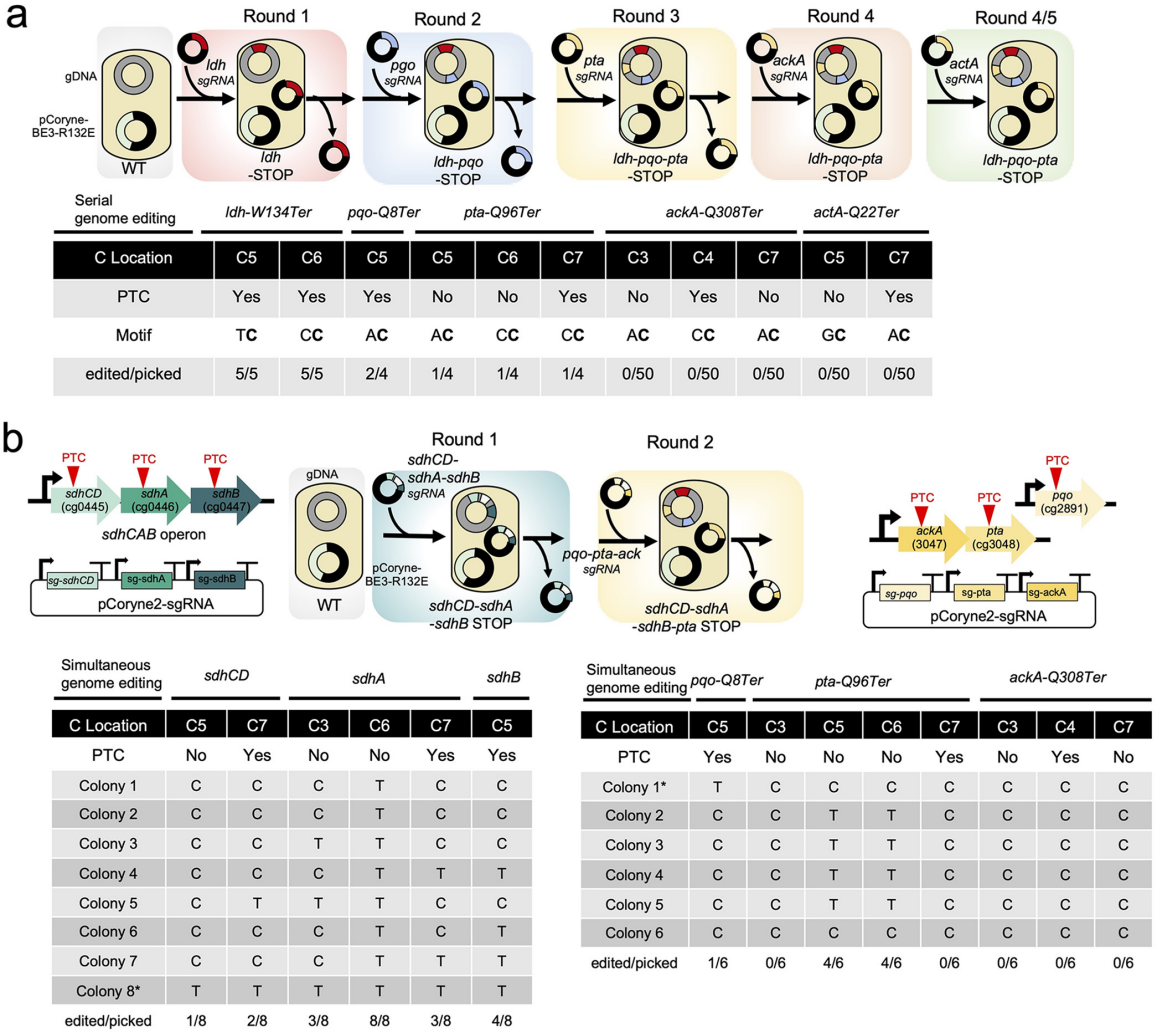
Multiplexed base editing by high-fidelity (HF) CBE. (a) Serial multiplex base editing using pCoryne-BE3-R132E (HF-CBE) and various sgRNA vectors targeting *ldh*, *pqo*, *pta*, *ackA*, or *actA* genes. For each round, competent C. glutamicum harboring pCoryne-BE3-R132E cells and a sgRNA vector were used for base editing. The sgRNA vectors were cured for the next rounds. Mutation analysis was shown for the C positions with the motif and mutation efficiencies (the numbers of edited colonies among picked colonies). (b) Simultaneous multiplex base editing using pCoryne-BE3-R132E (HF-CBE) and sgRNA vectors targeting both *sdhCD*, *sdhA*, and *sdhB* genes (round 1). The multiple targets for the second round were the *pqo*, *pta*, and *ackA* genes. Mutation analysis was shown for the C positions with mutation efficiencies (the numbers of edited colonies among picked colonies). Strains with asterisks were used for WGS analysis. PTC, premature termination codon.

Next, we sought to apply a HF-CBE-STOP to either serial or simultaneous MGE using a high-fidelity pCoryne-BE3-R132E for multiple gene knockouts. For the serial MGE, we started with the *ldh*-W134Ter-(HF) strain constructed above. After curing the sgRNA plasmids (ldh-sg134), we transferred a sgRNA vector targeting the *pqo*, *pta*, *ackA* and *act* gene, respectively. Each sgRNA was used for each round, and multiple gene knockouts were achieved to create triple mutants (strain *ldh-pqo-pta*) (Fig. 5a). Attempts to construct the mutants for rounds 4 and 5 failed to obtain the final desire mutant of PTCs (Fig. S7). This was due to the slightly lowered editing activity of pCoryne-BE3-R132E (Fig. 4d) rather than the original pCoryne-BE3 at less preferred target C positions in the editing window (Fig. 1c). As we studied earlier (Fig. S4), the serial MGE requires an increased number of the repetitive procedure corresponding to the total desired mutations.

To overcome the limitations of the serial MGE, simultaneous MGE was highly demanded to construct the desired mutants. We targeted the six gene knockouts: (i) the *sdhCD-sdhA-sdhB* operon in the first round and (ii) the *pta*-*ack* operon and the *pqo* gene in the second round (Fig. 5b). Each round utilized simultaneous expression of the triplet sgRNAs to create the PTCs. We selected eight different colonies and analyzed the C positions and C to T conversion mutations, resulting in one desired mutant of whole *sdhCDAB* operon inactivation (strain sdhCD-sdhA-sdhB). Then, the second round of simultaneous MGE was performed, yielding knockouts (KOs) of *pqo* (one correct colony over six picked colonies or *pta* (four edited mutations over six picked colonies). Inactivation of *ackA* failed due to the less favorable target C positions. Regardless of serial or simultaneous MGE, the mutation efficiency highly depends on the target C position to build the PTCs in the intrinsic DNA sequence of the target gene. Thus, a careful selection of the protospacer for CBE-STOP must be advised in a combination of target C position and motif. In the previous study, the CRISPR-cBEST targeting three genes in a single sgRNA and coexpressing Cys4 endoribonuclease has also successfully performed multiplexed genome engineering in S. coelicolor (19). Still, simultaneous multiplex genome engineering requires complex design principles of the sgRNA due to repetitive DNA units and efficacy of sgRNA processing. To avoid repetitive DNA sequences, nonrepetitive genetic parts of long sgRNA arrays for CRISPR-Cas systems could be useful for the next generation of multiplexed CRISPR-base editing (33).

### Genome-wide off-target effects for MGE using high-fidelity CBE (pCoryne-BE3-R132E).

**FIG 6:**
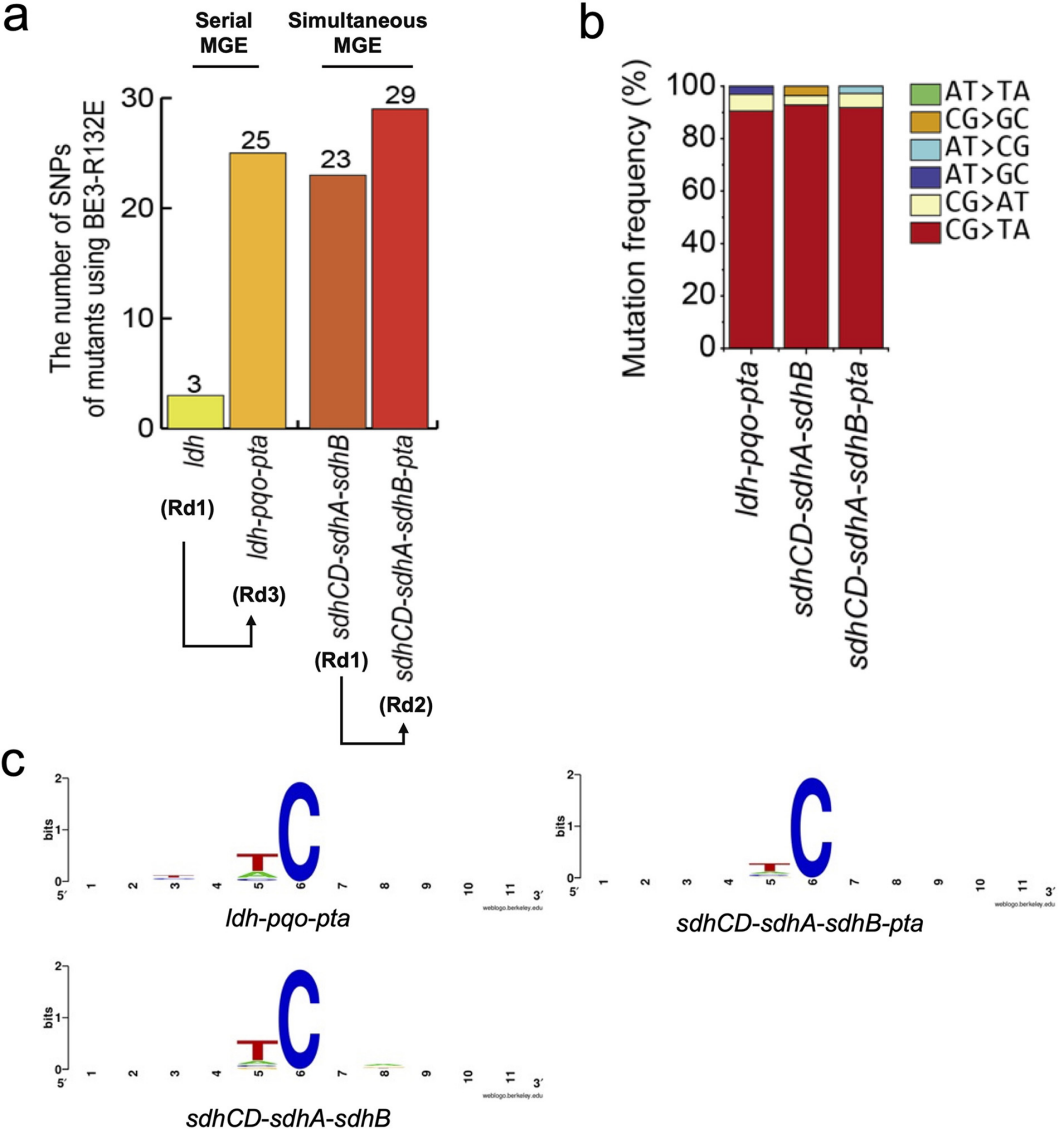
Genome-wide WGS analysis of the off-targets induced by HF-CBE (pCoryne-BE3-R132) for the multiplexed base editing. (a) The number of substitutions in *ldh*, *ldh-pqo-pta*, *sdhCD-sdhA-sdhB*, and *sdhCD-sdhA-sdhB-pta* mutants using pCoryne-BE3-R132E. WGS was duplicated. (b) Mutation frequencies (%) of the bases for those MGE mutants using pCoryne-BE3-R132E. (c) DNA logo for sequence context of the off-targets for those MGE mutants. The flanking sequences (2 bp on either side) were aligned at target cytosine positions. MGE, multiplexed genome editing; SNP, single-nucleotide polymorphism.

To investigate the genome-wide DNA off-target effect using a high-fidelity pCoryne-BE3-R132E for MGEs, we analyzed WGS of the C. glutamicum mutant strains developed in this study. For the serial MGE, WGS data showed that the total number of SNVs was slightly increased from round 1 (strain *ldh*; 3 SNVs) to round 3 (strain *ldh-pqo-pta*; 25 SNVs) (Fig. 6a; Table S11). Notably, 23 SNVs were identified in strain *sdhCD-sdhA-sdhB* at the first round of the simultaneous MGE. After the second MGE, an additional six SNVs were identified. Based on the high frequencies of C-to-T mutations, those SNVs were also believed to be off-target mutations generated by pCoryne-BE3-R132E regardless of sgRNAs (Fig. 6b). In addition, further analysis of the sequence contexts neighboring each SNV revealed strong preference for the target motif 5′-T**C** (Fig. 6c) as the promiscuous cytosine deamination activity of the pCoryne-BE3-R132E.

Compared to the off-target SNVs generated by MGE using the original pCoryne-BE3 (184 SNVs in BOL-1-STOP), either serial or simultaneous MGE using high-fidelity pCoryne-BE3-R132E generated significantly lower SNVs (25 SNVs in strain *ldh-pqo-pta*; 29 SNVs in strain *sdhCD-sdhA-sdhB-pta*). Interestingly, approximately 10 SNVs can be generated during each MGE using high-fidelity pCoryne-BE3-R132E. Then, simultaneous MGE has an advantage over serial MGE by shortening the total MGE procedures when more than two targets are demanded. Finally, pCoryne-BE3-R132E has been proven with high efficiency of the C-to-T mutations at the desired target and low off-target effects in the MGE for metabolic engineering of C. glutamicum.

## DISCUSSION

Microbial genome engineering requires precise genome editing tools to ensure construction of the microbial cell factories according to the design or to answer fundamental questions about the cellular process. Since CRISPR-guided genome engineering has accelerated the speed of the strain development at the desired mutations, the efficiency and single-base resolution precision in base editing as a genetic tool are the most crucial factors that must be considered before practical applications in the industry. In terms of the efficiency, two kinds of CBEs have been successfully applied to base editing in bacteria: Target-AID (d/nCas9-PmCDA) and BE3 (rAPOBEC1-nCas9-UGI). Compared with the Target-AID that showed high base editing frequencies (maximum 100% in a single-genome editing) at the C2 to C4 positions (20) and C-to-G by-products of base editing in some cases (13), counting the PAM as positions 21 to 23, the pCoryne-BE3 exhibited also high editing frequencies (maximum, 100%; average, 90.5%; *n* = 42; C6 position) consistently at the C4 to C7 positions that were similar to CRISPR-cBEST (a BE3 variant) and other BE3 systems (34). Unlike Target-AID, bacterial BE3 including pCoryne-BE3 in this study exhibited a strong 5′-TC-preference, which could be further engineered for broader applications.

Compared to the conventional genome engineering tools such as λ-Red-mediated one-step inactivation (7) and SacB-mediated HR (5), the CBE-STOP tool showed a short hands-on procedure in which only one-step colony selection was needed, whereas those conventional methods require a two-step selection of antibiotics selection and counterselection. Regardless of genetic tools, desired foreign DNA templates must be prepared before performing genome engineering. λ-Red-mediated recombination requires simple PCR steps to generate a linear dsDNA using the plasmid template and genomic DNA. However, SacB-mediated recombination needs a target plasmid that contains two separate homologous regions with desired foreign DNA templates, which requires conventional gene cloning work. In the case of CBE-STOP, an oligonucleotide having a proper protospacer can be easily synthesized and used for simple sgRNA cloning. These features may shorten the total handling time and will be useful for automated genome engineering (20).

Second, no DNA scar remained in the final mutant using the CBE-STOP. However, 82 to 85 nt of flippase recognition target sites as the scar was left in case of λ-Red-mediated one-step inactivation. This could be problematic in multiplexed genome engineering due to undesired recombination. Like CBE-STOP, SacB-mediated HR does not leave a DNA scar at the end, but the mutation frequencies were considerably lower (55% mutant; 45% wild-type) than CBE-STOP (average 90.5%; this study) and λ-Red-mediated recombination (maximum knockout efficiency of 100% for some open reading frames [ORFs]) (35). Theoretically, SacB-mediated recombination does not limit the design principle to generate the desired mutations from either single-nucleotide exchange to kilobase-size DNA deletion. However, CBE-STOP can only edit a single nucleotide with a C-to-T conversion. Depending on the target sequences, various base editors with different versions of Cas9 and Cas12a may be developed to generate specific and successful mutations (34). Recent prime editing that enables all base-to-base conversions without requiring DSBs or donor template can be applied to various industrial bacteria (36, 37), and an optimized prime editing technology will be developed for species-specific industrial hosts, including C. glutamicum. So far, useful CRISPR software for base editing is available (38). Further development will lower the barrier of the sgRNA design considerations of PAM site, editing windows, and target motif preference to achieve broader applications of genome engineering.

Increasing a single-base resolution precision in base editing can be achieved by both reducing genome-wide off-targets and narrowing the editing windows. To ensure the high-fidelity precision of the BE3 by reducing off-targets, we identified genome-wide off-targets (48 SNVs) for single-gene mutations in C. glutamicum. A pCoryne-BE3 induced the gRNA-independent off-targets in the absence of any selection pressure, which was consistent with previous results by the Weber and Lee groups showing 27 to 56 SNVs in S. coelicolor using CRISPR-cBEST (rAPOBEC1-nCas9-UGI) (19). We further found that 184 SNVs accumulated during multiple rounds of base editing, but HF-CBEs (pCoryne-BE3-R132E and YE1-BE3) substantially decreased the SNVs to three or eight, respectively. After MGE using pCoryne-BE3-R132E, the number of the off-target SNVs were also significantly reduced, compared to the original CBEs. Consistently, two-cell embryo injection (GOTI) experiments (21) by the Yang group revealed that an average of 283 SNVs was observed in BE3-treated mouse embryos, but this decreased 20-fold in BE3-R132E- and YE1-BE3-treated samples (24).

A previous study based on Target-AID by the Kondo group revealed that WGS data of rifampicin (Rif)-selected E. coli strains with nontargeting sgRNA identified two SNVs, and SNVs increased during multiplexed editing (18). Although the number of SNVs induced by Target-AID was lower than that induced by pCoryne-BE3 and comparable to HF-CBEs, further studies under the same experimental conditions, such as the same organism without any selection pressures, as in our study, will be necessary for an exact comparison of the tools. The Ma group demonstrated Target-AID for genome engineering in C. glutamicum with a maximum of 100% editing performance (20). Subsequently, WGS showed that 18 SNVs from five single-gene edited strains were identified, and 13 SNVs were revealed as C•G-to-T•A substitutions, which could be regarded as sgRNA-independent off-targets by the Target-AID.

To increase the precision of the BE3 by narrowing the editing windows, it was necessary to discriminate the targeted C nucleotide in the window from the neighboring C nucleotides within one or two bases. Based on the YE1-BE3 (32), which has been successfully used for increasing precision in human cells, we also observed the narrower editing windows with YE1-BE3. However, its on-target activities at each C position were slightly less efficient than BE3-R132E. Regardless of modulating windows, pCoryne-BE3-R132E provided the precise genome editing tool for CBE-STOP in C. glutamicum without undesired off-targets.

In conclusion, we successfully engineered C. glutamicum by generating PTCs in the gene of interest via CBEs without requiring a foreign DNA temple and DSBs and constructed several single gene-inactivated strains, as well as a five-gene-inactivated strain, BOL-1-STOP. Despite the desired phenotype, the WGS data revealed that genome-wide substitution mutations occurred in each strain and further accumulated according to the duration time of CBEs; i.e., the BOL-1-STOP strain has about 4.5 times as many mutations as the *ldh*-STOP strain. Deep analysis of the mutations showed that (i) most mutations were C-to-T conversions, (ii) flanking sequences exhibited 5′-TCN-3′ sequences, and (iii) mutations were preferably positioned within the promoter regions, which tend to unwind during transcription or duplication. Taken together, we concluded that those mutations are derived by direct deaminase activity of CBEs regardless of the guide RNA sequences. To reduce such mutations, we adopted HF-CBEs (YE1-BE3 and BE3-R132E) for single-genome editing and MGE and revealed that the genome-wide mutations were drastically decreased, compared to original CBE (BE3). DNA base editing tools have undoubtedly advanced the field of microbial genome engineering, but they are not impeccable (39) to promote developing microbial cell factories. This report will facilitate further microbial genome engineering using precise cytosine base editing.

## MATERIALS AND METHODS

### Bacterial strains and growth conditions.

All bacterial strains and plasmids used in this study are listed in Table 1. E. coli DH5α (40) and C. glutamicum ATCC 13032 (wild-type) were used in this study. E. coli strains were grown in lysogeny broth (LB) medium (10 g/liter tryptone, 5 g/liter yeast extract, and 5 g/liter NaCl) at 37°С on a rotary shaker at 200 rpm. When appropriate, the medium was supplemented with 30 μg/mL chloramphenicol (Cm), 50 μg/mL kanamycin (Km), or 100 μg/mL spectinomycin (Spc). C. glutamicum ATCC 13032 and its derivatives were cultivated in brain-heart infusion broth supplemented with 91 g/liter sorbitol (BHIS) medium at 30°С on a rotary shaker at 120 rpm (41). When appropriate, the medium was supplemented with 7.5 μg/mL chloramphenicol, 25 μg/mL kanamycin, and/or 50 μg/mL spectinomycin. For flask cultivation, C. glutamicum strains were precultivated in BHIS medium overnight and then incubated aerobically in CgXII defined medium (50 mL media in a 250-mL baffled Erlenmeyer flask) containing 2% (wt/vol) or 4% (wt/vol) glucose as the sole carbon source, at 30°C on a rotary shaker at 120 rpm.

### Plasmid construction and transformation.

CBE-STOP requires a two-plasmid system (pCoryne-BE3/BE1 and pCoryne2-sgRNA) in C. glutamicum (Table 1). To construct pCoryne-BE1 or pCoryne-BE3, the plasmids for cytosine base editors were purchased from Addgene (Watertown, MA; BE1, catalog no. 73018; BE3, catalog no. 87437), and the base editing protein-coding regions (rAPOBEC1-XTEN-dCas9 for BE1 or rAPOBEC1-XTEN-nCas9-UGI for BE3) were cloned into a pBbEB2c-RFP (CoryneBrick vector) (42), yielding either pCoryne-BE1 or pCoryne-BE3, respectively. For high-fidelity CBE (24), pCoryne-YE1-BE3 and pCoryne-BE3-R132E were constructed by introducing point mutations into rAPOBEC1 (W90Y and R126E) and rAPOBEC1 (R132E) of the BE3 in pCoryne-BE3, respectively. pCoryne2-sgRNA was constructed by replacing the crRNA region in pJYS2_crtYF with the sgRNA region from pgRNA-bacteria using the BioBrick cloning method (EcoRI/BamHI) (26, 43) and was used as a parental sgRNA expression vector. Target sgRNAs for CBE-STOP were obtained by self-ligating the PCR products from pCoryne2-sgRNA using a forward primer containing a target-specific protospacer region (Table S2) and a universal reverse primer in association with the robotic platform at the SKy Biofoundry (Sungkyunkwan University, South Korea). For serial MGE, each target sgRNA was cloned into pgRNA-bacteria. For simultaneous MGE, multiple sgRNAs were cloned into the pCoryne2-AarI vector using AarI, which is one of the type IIs enzymes. pCoryne2-AarI was obtained by self-ligating the PCR products from pCoryne2-sgRNA using forward and reverse primers, including AarI and EcoRI recognition sites. The oligonucleotide primers used for cloning are shown in Table S2. The resulting plasmids were introduced into C. glutamicum strains by electroporation, and strain validation was performed using colony PCR (41, 44).

### Genome-wide analysis of PAM sites for CBE-STOP.

A web-based designer and analysis tool for CRISPR base editing, BE-Designer, was used to generate the genome-wide base editing PAMs and the CRISPR-STOP PAMs for C. glutamicum ATCC 13032 (NC_003450.3) (38). The CBE-STOP can create a C•G-to-T•A mutation within the editable ranges of the codons of CAA (Gln), CAG (Gln), CGA (Arg), or TGG (Trp) as the C4 to C8 positions of the PAM sequence (5′-NGG), generating the STOP codons (TAA, TAG, and TGA) in the coding strand. Thus, all CBE-STOP PAMs among the genome-wide base editing PAMs in C. glutamicum were identified, and the corresponding protospacers were used for cloning (Table 1). For the NG-PAM sequence (5′-NG), the CBE-STOP PAMs were expanded with the same editing-window positions to create STOP codons.

### Construction of nonsense mutant using CBE-STOP and plasmid curing.

To perform CBE-STOP in C. glutamicum, a two-plasmid system was employed (pCoryne-BE3/BE1 and pCoryne2-sgRNA). First, a pCoryne-BE1/BE3 plasmid was transferred to the parental C. glutamicum strain (WT in this study). After competent C. glutamicum strains harboring pCoryne-BE1/BE3 were prepared in the presence of anhydrotetracycline (aTc, 100 nM) as an inducer, pCoryne2-sgRNAs were transferred for base editing by electroporation (41). Colonies were grown on BHIS agar plates in the presence of both antibiotics and aTc (200 nM), and strain validation was performed using colony PCR and DNA sequencing. Plasmid curing of either pCoryne-BE3 or pCoryne2-sgRNAs was performed by cultivation in liquid antibiotic-free medium (BHIS) at 30°С for at least 2 days. Loss of the corresponding plasmids was confirmed on antibiotic-containing agar plates. To construct a succinate producer analog (BOL-1-STOP), sequential transformation was performed with the next round of sgRNAs after curing the previous sgRNA (Fig. S4).

### High-performance liquid chromatography analysis to measure metabolites.

Glucose and organic acids (lactate, acetate, and succinate) in the supernatant were quantified using high-performance liquid chromatography (HPLC) as described previously (44). Briefly, the culture supernatant was passed through a syringe filter (pore size, 0.45 μm). The concentrations of glucose and organic acids were detected by HPLC (Agilent 1260, Agilent Technologies, Waldbronn, Germany) equipped with a refractive index detector (RID) and a Hi-Plex H, 7.7 × 300-mm column (Agilent Technologies) under the following conditions: sample volume of 20 μL, mobile phase of 5 mM H_2_SO_4_, a flow rate of 0.6 mL/min, and column temperature of 65°C.

### Production of biomass for anaerobic succinate production.

A total of 5 mL of BHIS medium were inoculated with a single colony of the desired C. glutamicum from a fresh BHI agar plate, and the culture was incubated on a rotary shaker for 16 h at 30°C. Subsequently, the cells were used to inoculate a 250 mL baffled shake flask with 50 mL of CGXII medium containing 222 mM glucose. Once the optimal density at 600 nm (OD_600_) reached 12, the culture was harvested, and the cell pellet was washed with CgXII medium without a carbon source and transferred to 50 mL CGXII medium supplemented with 222 mM glucose and 200 mM NaHCO_3_ in a 250-mL airtight nonbaffled shake flask with a screw cap. The flask containing the medium was purged with nitrogen gas before cell inoculation. The anaerobic culture was incubated on a rotary shaker for 16 h at 30°C and 120 rpm.

### Whole-genome sequencing analysis using PacBio or Illumina.

From randomly picked colonies on the BHIS-agar plate, edited C. glutamicum strains were incubated overnight at 30°C and 200 rpm in 3 mL BHIS medium. Genomic DNA samples were extracted using the Wizard Genomic DNA purification kit (Promega, Madison, WI, USA) according to the manufacturer’s instructions. WGS analysis using PacBio was performed by ChunLab, Inc. (Seoul, South Korea). The NGS library was prepared using the FastDNA spin kit for soil (MP Biomedicals, Irvine, CA, USA). Quality check was performed using the Quant-iT PicoGreen dsDNA assay kit (Invitrogen, Carlsbad, CA, USA). The prepared library was sequenced using the PacBio platform. Genomic DNA samples were subjected to quality control using the PacBio 20 K method. Sequencing depth was 145.24 × coverage of the genome, which was assembled into one contig with the EzBioCloud. In parallel, WGS analysis using Illumina was performed at Macrogen (Seoul, South Korea). The NGS library was produced using a TruSeq Nano DNA kit (Illumina, San Diego, CA, USA) after DNA fragmentation. The library was sequenced using the Illumina platform. The quality of NGS results was confirmed by FastQC (http://www.bioinformatics.babraham.ac.uk/projects/fastqc). Assembly sequences were produced using SPAdes (http://cab.spbu.ru/software/spades/), and the quality was assessed using BUSCO (https://busco.ezlab.org/). Assembly sequences were aligned using bwa mem (http://bio-bwa.sourceforge.net/bwa.shtml). The BAM and SAM files were arranged using the same tools (http://www.htslib.org/). The annotation of substitution was analyzed with the NC_003450 GenBank file and available data for C. glutamicum ATCC 13032 (28). All additional analyses were performed using Python (https://github.com/Gue-ho/CoryneAnalysis). All WGSs in this study were performed in duplicate.

### Deep sequencing of the target regions.

Genomic DNA samples prepared from WGS analysis were used for deep sequencing. The primer oligonucleotides for PCR (Table S10) were designed to include off-targets and were synthesized by Macrogen. The PCR products were amplified by two-step PCR using SUN *Taq* DNA polymerase (SUN Genetics, Daejeon, South Korea). The libraries were purified using a PCR purification kit (Expin PCR SV mini, 200p; GeneAll, Gyeonggi, South Korea) and were sequenced using a MiniSeq mid output kit with a TruSeq HT dual index system (Illumina). The NGS results were analyzed to confirm the off-targets in the NGS file using Python.

### Data availability.

The data supporting the findings of this study are available in the article and its supplemental material. Additional data are available from the corresponding author upon reasonable request. The strains and plasmids developed in this study can be provided only for noncommercial purposes. Whole-genome sequencing data via Illumina-seq and PacBio-seq have been deposited in the NCBI Sequence Read Archive database under accession number PRJNA718416. Source codes for WGS and deep sequencing analyses are available at https://github.com/Gue-ho/CoryneAnalysis.

## References

[1] Ajikumar PK, Xiao WH, Tyo KEJ, Wang Y, Simeon F, Leonard E, Mucha O, Phon TH, Pfeifer B, Stephanopoulos G. 2010. Isoprenoid pathway optimization for taxol precursor overproduction in *Escherichia coli*. Science 330:70–74. doi:10.1126/science.1191652. 20929806 PMC3034138

[2] Choi YJ, Lee SY. 2013. Microbial production of short-chain alkanes. Nature 502:571–574. doi:10.1038/nature12536. 24077097

[3] Luo X, Reiter MA, d’Espaux L, Wong J, Denby CM, Lechner A, Zhang Y, Grzybowski AT, Harth S, Lin W, Lee H, Yu C, Shin J, Deng K, Benites VT, Wang G, Baidoo EEK, Chen Y, Dev I, Petzold CJ, Keasling JD. 2019. Complete biosynthesis of cannabinoids and their unnatural analogues in yeast. Nature 567:123–126. doi:10.1038/s41586-019-0978-9. 30814733

[4] Riglar DT, Silver PA. 2018. Engineering bacteria for diagnostic and therapeutic applications. Nat Rev Microbiol 16:214–225. doi:10.1038/nrmicro.2017.172. 29398705

[5] Schafer A, Tauch A, Jager W, Kalinowski J, Thierbach G, Puhler A. 1994. Small mobilizable multi-purpose cloning vectors derived from the *Escherichia coli* plasmids pK18 and pK19: selection of defined deletions in the chromosome of *Corynebacterium glutamicum*. Gene 145:69–73. doi:10.1016/0378-1119(94)90324-7. 8045426

[6] Li X-t, Thomason LC, Sawitzke JA, Costantino N, Court DL. 2013. Positive and negative selection using the tetA-sacB cassette: recombineering and P1 transduction in *Escherichia coli*. Nucleic Acids Res 41:e204. doi:10.1093/nar/gkt1075. 24203710 PMC3905872

[7] Datsenko KA, Wanner BL. 2000. One-step inactivation of chromosomal genes in *Escherichia coli* K-12 using PCR products. Proc Natl Acad Sci USA 97:6640–6645. doi:10.1073/pnas.120163297. 10829079 PMC18686

[8] Yu BJ, Kang KH, Lee JH, Sung BH, Kim MS, Kim SC. 2008. Rapid and efficient construction of markerless deletions in the *Escherichia coli* genome. Nucleic Acids Res 36:e84. doi:10.1093/nar/gkn359. 18567910 PMC2504295

[9] Jiang W, Bikard D, Cox D, Zhang F, Marraffini LA. 2013. RNA-guided editing of bacterial genomes using CRISPR-Cas systems. Nat Biotechnol 31:233–239. doi:10.1038/nbt.2508. 23360965 PMC3748948

[10] Selle K, Barrangou R. 2015. Harnessing CRISPR-Cas systems for bacterial genome editing. Trends Microbiol 23:225–232. doi:10.1016/j.tim.2015.01.008. 25698413

[11] Li Y, Lin Z, Huang C, Zhang Y, Wang Z, Tang Y-J, Chen T, Zhao X. 2015. Metabolic engineering of *Escherichia coli* using CRISPR-Cas9 meditated genome editing. Meta Eng 31:13–21. doi:10.1016/j.ymben.2015.06.006. 26141150

[12] Jiang Y, Qian F, Yang J, Liu Y, Dong F, Xu C, Sun B, Chen B, Xu X, Li Y, Wang R, Yang S. 2017. CRISPR-Cpf1 assisted genome editing of *Corynebacterium glutamicum*. Nat Commun 8:15179. doi:10.1038/ncomms15179. 28469274 PMC5418603

[13] Nishida K, Arazoe T, Yachie N, Banno S, Kakimoto M, Tabata M, Mochizuki M, Miyabe A, Araki M, Hara KY, Shimatani Z, Kondo A. 2016. Targeted nucleotide editing using hybrid prokaryotic and vertebrate adaptive immune systems. Science 353:aaf8729. doi:10.1126/science.aaf8729. 27492474

[14] Komor AC, Kim YB, Packer MS, Zuris JA, Liu DR. 2016. Programmable editing of a target base in genomic DNA without double-stranded DNA cleavage. Nature 533:420–424. doi:10.1038/nature17946. 27096365 PMC4873371

[15] Kuscu C, Parlak M, Tufan T, Yang J, Szlachta K, Wei X, Mammadov R, Adli M. 2017. CRISPR-STOP: gene silencing through base-editing-induced nonsense mutations. Nat Methods 14:710–712. doi:10.1038/nmeth.4327. 28581493

[16] Billon P, Bryant EE, Joseph SA, Nambiar TS, Hayward SB, Rothstein R, Ciccia A. 2017. CRISPR-mediated base editing enables efficient disruption of eukaryotic genes through induction of STOP codons. Mol Cell 67:1068–1079.e4. doi:10.1016/j.molcel.2017.08.008. 28890334 PMC5610906

[17] Wang Y, Liu Y, Zheng P, Sun J, Wang M. 2021. Microbial base editing: a powerful emerging technology for microbial genome engineering. Trends Biotechnol 39:165–180. doi:10.1016/j.tibtech.2020.06.010. 32680590

[18] Banno S, Nishida K, Arazoe T, Mitsunobu H, Kondo A. 2018. Deaminase-mediated multiplex genome editing in *Escherichia coli*. Nat Microbiol 3:423–429. doi:10.1038/s41564-017-0102-6. 29403014

[19] Tong Y, Whitford CM, Robertsen HL, Blin K, Jørgensen TS, Klitgaard AK, Gren T, Jiang X, Weber T, Lee SY. 2019. Highly efficient DSB-free base editing for streptomycetes with CRISPR-BEST. Proc Natl Acad Sci USA 116:20366–20375. doi:10.1073/pnas.1913493116. 31548381 PMC6789908

[20] Wang Y, Liu Y, Liu J, Guo Y, Fan L, Ni X, Zheng X, Wang M, Zheng P, Sun J, Ma Y. 2018. MACBETH: multiplex automated *Corynebacterium glutamicum* base editing method. Metab Eng 47:200–210. doi:10.1016/j.ymben.2018.02.016. 29580925

[21] Zuo E, Sun Y, Wei W, Yuan T, Ying W, Sun H, Yuan L, Steinmetz LM, Li Y, Yang H. 2019. Cytosine base editor generates substantial off-target single-nucleotide variants in mouse embryos. Science 364:289–292. doi:10.1126/science.aav9973. 30819928 PMC7301308

[22] Jin S, Zong Y, Gao Q, Zhu Z, Wang Y, Qin P, Liang C, Wang D, Qiu J-L, Zhang F, Gao C. 2019. Cytosine, but not adenine, base editors induce genome-wide off-target mutations in rice. Science 364:292–295. doi:10.1126/science.aaw7166. 30819931

[23] Grünewald J, Zhou R, Garcia SP, Iyer S, Lareau CA, Aryee MJ, Joung JK. 2019. Transcriptome-wide off-target RNA editing induced by CRISPR-guided DNA base editors. Nature 569:433–437. doi:10.1038/s41586-019-1161-z. 30995674 PMC6657343

[24] Zuo E, Sun Y, Yuan T, He B, Zhou C, Ying W, Liu J, Wei W, Zeng R, Li Y, Yang H. 2020. A rationally engineered cytosine base editor retains high on-target activity while reducing both DNA and RNA off-target effects. Nat Methods 17:600–604. doi:10.1038/s41592-020-0832-x. 32424272

[25] Nishimasu H, Shi X, Ishiguro S, Gao L, Hirano S, Okazaki S, Noda T, Abudayyeh OO, Gootenberg JS, Mori H, Oura S, Holmes B, Tanaka M, Seki M, Hirano H, Aburatani H, Ishitani R, Ikawa M, Yachie N, Zhang F, Nureki O. 2018. Engineered CRISPR-Cas9 nuclease with expanded targeting space. Science 361:1259–1262. doi:10.1126/science.aas9129. 30166441 PMC6368452

[26] Park J, Shin H, Lee SM, Um Y, Woo HM. 2018. RNA-guided single/double gene repressions in *Corynebacterium glutamicum* using an efficient CRISPR interference and its application to industrial strain. Microb Cell Fact 17:4. doi:10.1186/s12934-017-0843-1. 29316926 PMC5759794

[27] Litsanov B, Brocker M, Bott M. 2012. Toward homosuccinate fermentation: metabolic engineering of *Corynebacterium glutamicum* for anaerobic production of succinate from glucose and formate. Appl Environ Microbiol 78:3325–3337. doi:10.1128/AEM.07790-11. 22389371 PMC3346441

[28] Pfeifer-Sancar K, Mentz A, Rückert C, Kalinowski J. 2013. Comprehensive analysis of the *Corynebacterium glutamicum* transcriptome using an improved RNAseq technique. BMC Genomics 14:888. doi:10.1186/1471-2164-14-888. 24341750 PMC3890552

[29] Yu Y, Leete TC, Born DA, Young L, Barrera LA, Lee S-J, Rees HA, Ciaramella G, Gaudelli NM. 2020. Cytosine base editors with minimized unguided DNA and RNA off-target events and high on-target activity. Nat Commun 11:2052. doi:10.1038/s41467-020-15887-5. 32345976 PMC7189382

[30] Liu LD, Huang M, Dai P, Liu T, Fan S, Cheng X, Zhao Y, Yeap L-S, Meng F-L. 2018. Intrinsic nucleotide preference of diversifying base editors guides antibody *ex vivo* affinity maturation. Cell Rep 25:884–892.e3. doi:10.1016/j.celrep.2018.09.090. 30355495

[31] Doman JL, Raguram A, Newby GA, Liu DR. 2020. Evaluation and minimization of Cas9-independent off-target DNA editing by cytosine base editors. Nat Biotechnol 38:620–628. doi:10.1038/s41587-020-0414-6. 32042165 PMC7335424

[32] Kim YB, Komor AC, Levy JM, Packer MS, Zhao KT, Liu DR. 2017. Increasing the genome-targeting scope and precision of base editing with engineered Cas9-cytidine deaminase fusions. Nat Biotechnol 35:371–376. doi:10.1038/nbt.3803. 28191901 PMC5388574

[33] Reis AC, Halper SM, Vezeau GE, Cetnar DP, Hossain A, Clauer PR, Salis HM. 2019. Simultaneous repression of multiple bacterial genes using nonrepetitive extra-long sgRNA arrays. Nat Biotechnol 37:1294–1301. doi:10.1038/s41587-019-0286-9. 31591552

[34] Rees HA, Liu DR. 2018. Base editing: precision chemistry on the genome and transcriptome of living cells. Nat Rev Genet 19:770–788. doi:10.1038/s41576-018-0059-1. 30323312 PMC6535181

[35] Baba T, Ara T, Hasegawa M, Takai Y, Okumura Y, Baba M, Datsenko KA, Tomita M, Wanner BL, Mori H. 2006. Construction of *Escherichia coli* K-12 in-frame, single-gene knockout mutants: the Keio collection. Mol Syst Biol 2:2006.0008. doi:10.1038/msb4100050. PMC1681482 16738554

[36] Anzalone AV, Randolph PB, Davis JR, Sousa AA, Koblan LW, Levy JM, Chen PJ, Wilson C, Newby GA, Raguram A, Liu DR. 2019. Search-and-replace genome editing without double-strand breaks or donor DNA. Nature 576:149–157. doi:10.1038/s41586-019-1711-4. 31634902 PMC6907074

[37] Tong Y, Jørgensen TS, Whitford CM, Weber T, Lee SY. 2021. A versatile genetic engineering toolkit for *E. coli* based on CRISPR-prime editing. Nat Commun 12:5206. doi:10.1038/s41467-021-25541-3. 34471126 PMC8410854

[38] Hwang G-H, Park J, Lim K, Kim S, Yu J, Yu E, Kim S-T, Eils R, Kim J-S, Bae S. 2018. Web-based design and analysis tools for CRISPR base editing. BMC Bioinformatics 19:542. doi:10.1186/s12859-018-2585-4. 30587106 PMC6307267

[39] Jeong YK, Song B, Bae S. 2020. Current status and challenges of DNA base editing tools. Mol Ther 28:1938–1952. doi:10.1016/j.ymthe.2020.07.021. 32763143 PMC7474268

[40] Hanahan D. 1983. Studies on transformation of *Escherichia coli* with plasmids. J Mol Biol 166:557–580. doi:10.1016/s0022-2836(83)80284-8. 6345791

[41] Eggeling L, Bott M. 2005. Handbook of Corynebacterium glutamicum. CRC Press, Boca Raton, FL.

[42] Kang MK, Lee J, Um Y, Lee TS, Bott M, Park SJ, Woo HM. 2014. Synthetic biology platform of CoryneBrick vectors for gene expression in *Corynebacterium glutamicum* and its application to xylose utilization. Appl Microbiol Biotechnol 98:5991–6002. doi:10.1007/s00253-014-5714-7. 24706215

[43] Qi LS, Larson MH, Gilbert LA, Doudna JA, Weissman JS, Arkin AP, Lim WA. 2013. Repurposing CRISPR as an RNA-guided platform for sequence-specific control of gene expression. Cell 152:1173–1183. doi:10.1016/j.cell.2013.02.022. 23452860 PMC3664290

[44] Yoon J, Woo HM. 2018. CRISPR interference-mediated metabolic engineering of *Corynebacterium glutamicum* for homo-butyrate production. Biotechnol Bioeng 115:2067–2074. doi:10.1002/bit.26720. 29704438

[45] Kang M-K, Eom J-H, Kim Y, Um Y, Woo HM. 2014. Biosynthesis of pinene from glucose using metabolically-engineered *Corynebacterium glutamicum*. Biotechnol Lett 36:2069–2077. doi:10.1007/s10529-014-1578-2. 24930112

[46] Rees HA, Komor AC, Yeh W-H, Caetano-Lopes J, Warman M, Edge ASB, Liu DR. 2017. Improving the DNA specificity and applicability of base editing through protein engineering and protein delivery. Nat Commun 8:15790. doi:10.1038/ncomms15790. 28585549 PMC5467206

