# Supplementary material for: High-Fidelity Cytosine Base Editing in a GC-Rich Corynebacterium glutamicum with Reduced DNA Off-Target Editing Effects
Source: Microbiol Spectr. 2022 Nov 14;10(6):e03760-22. doi: 10.1128/spectrum.03760-22 (PMC9769817; doi:10.1128/spectrum.03760-22)
Supplement: Supplemental file 1 — Supplemental material. Download spectrum.03760-22-s0001.pdf, PDF file, 4.0 MB [file spectrum.03760-22-s0001.pdf]

Supplementary information

High-fidelity cytosine editing in a GC-rich *Corynebacterium glutamicum* with reduced DNA off-target editing effects

Yu Been Heo[1,2,#], Gue-Ho Hwang[3,#], Seok Won Kang[1], Sangsu Bae[4,*], Han Min Woo[1,2,*]

[1]Department of Food Science and Biotechnology, Sungkyunkwan University (SKKU), 2066 Seobu-ro, Jangan-gu, Suwon 16419, Republic of Korea

[2]BioFoundry Research Center, Institute of Biotechnology and Bioengineering, Sungkyunkwan University (SKKU), 2066 Seobu-ro, Jangan-gu, Suwon 16419, Republic of Korea

[3]Department of Chemistry, Hanyang University, 222 Wangsimni-ro, Seongdong-gu, Seoul 04763, Republic of Korea

[4]Department of Biomedical Sciences, Department of Biochemistry and Molecular Biology, Seoul National University College of Medicine, 103 Daehak-ro, Jongno-gu, Seoul 03080, Republic of Korea

Table of Contents

Figure S1. *In silico* investigation of applicable base editing targets using CBE-STOP.

Figure S2. Comparison of editing activities of BE1 and BE3 in *C. glutamicum*.

Figure S3. Nonsense mutations in different genes in *C. glutamicum* using CBE-STOP.

Figure S4. Comparison of the procedures for multiplexed gene editing using CBE-STOP in *C. glutamicum*.

Figure S5. Sequence verification and evaluation of BOL-1-STOP strain.

Figure S6. Cell growth and lactate production of WT and *ldh*-W134Ter strains using either BE3-R132E or YE1-BE3.

Figure S7. Attempts to perform the HF-CBE-STOP for *ackA* and *actA* genes using BE3-R132E.

Table S1. All possible BE3-STOPs using either NGG-CBE or NG-CBE.

Table S2. Oligonucleotides used for gene cloning.

Table S3. Target protospacer used for CBE-STOP.

Table S4. List of the mutation frequencies using CBE-STOP.

Table S5. List of the sequence-context-dependent motifs and base editing performance using CBE-STOP.

Table S6. List of the off-targets for ldh-W134Ter and BOL-1-STOP samples using CBE-STOP.

Table S7. List of off-targets for *ldh*-W134Ter using HF-CBE-STOP.

Table S8. List of the target genes and their target protospacers. Base editing efficiencies using HF-CBE-STOP.

Table S9. List of the sequence-context-dependent motifs and base editing performance using HF-CBE-STOP.

Table S10. List of the primers for deep sequencing.

Table S11. List of the off-targets identified through MGEs using pCoryne-BE3-R132E

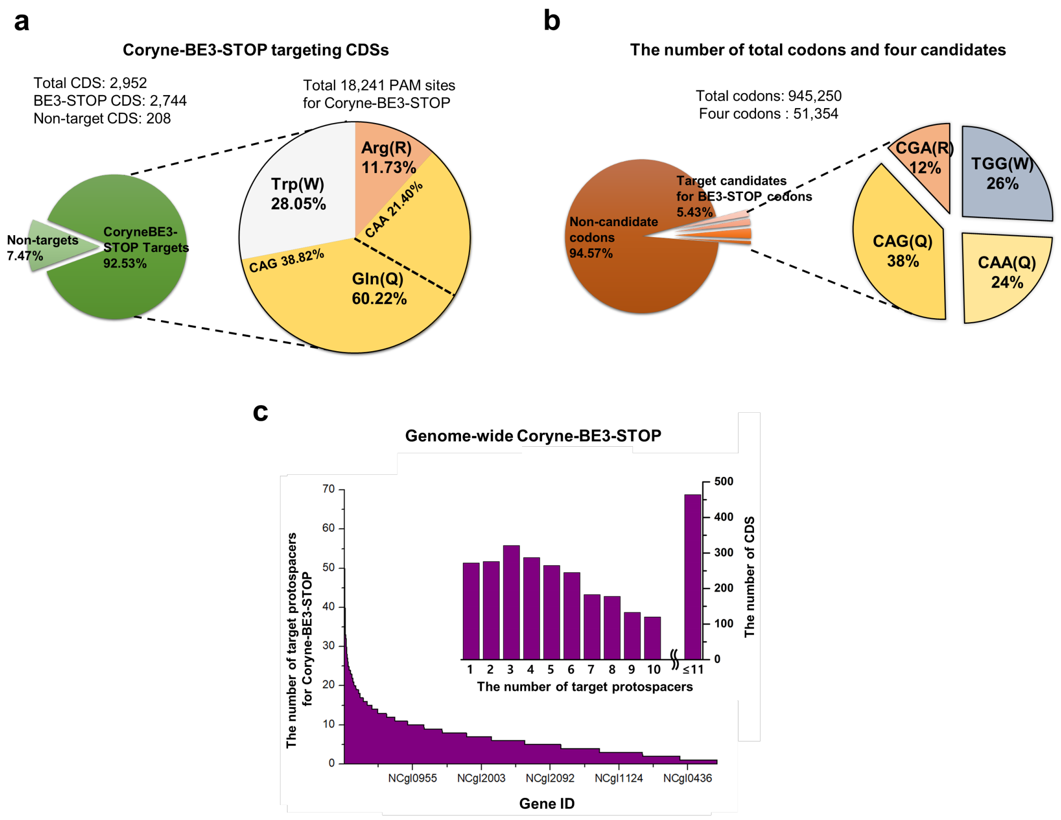

Figure S1. *In silico* investigation of applicable base editing targets using CBE-STOP. (**A**) Analysis of the number of targetable genes dependent on PAM sites (5′-NGG) on *C. glutamicum* genome for using CBE-STOP and ratio of targetable amino acids on targetable CDS on *C. glutamicum* to introduce STOP codon by CBE-STOP (**B**) Ratio of targetable amino acids on entire *C. glutamicum* genome and relative composition ratio of each amino acid among them. (**C**) Number of targetable protospacers with BE3 on *C. glutamicum* genome considering 5′-NGG PAM and target C location in C4–C8 of protospacer (PAM; C21–C23)

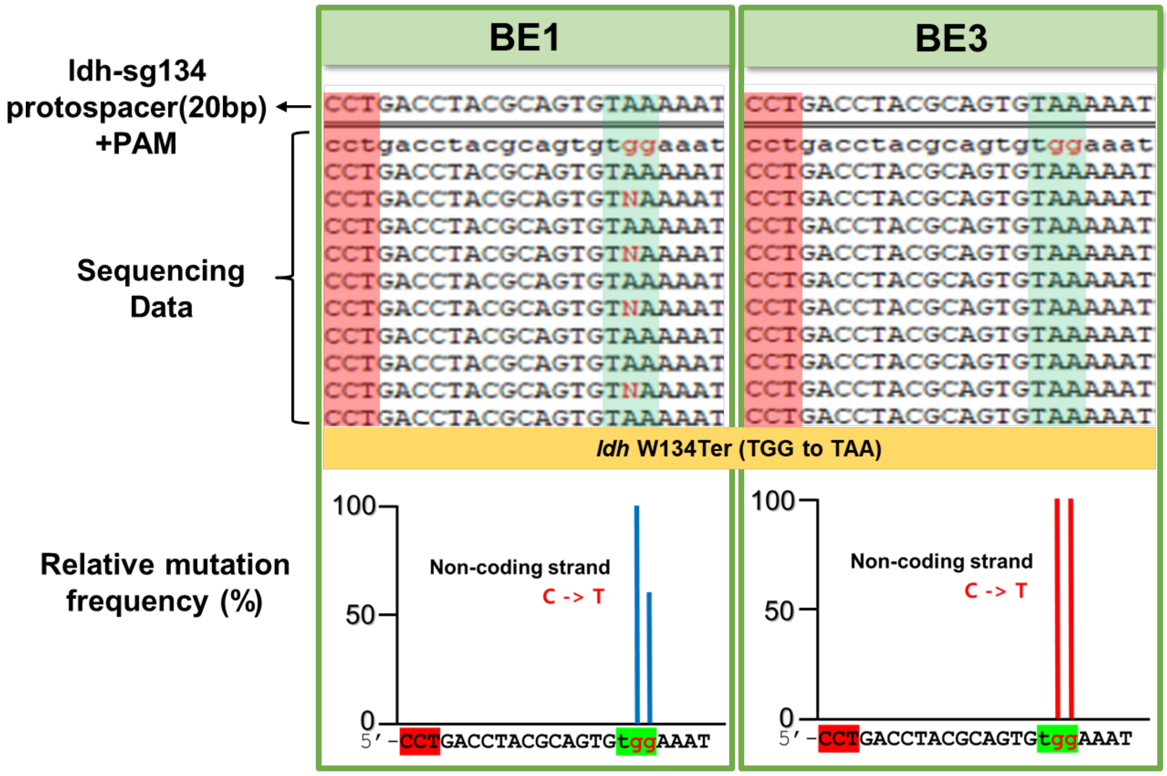

	BE1		BE3	
Base editing efficiency	C location	Editing efficiency (%)	C location	Editing efficiency (%)
	C5	60	C5	100
	C6	100	C6	100
STOP efficiency (%)	100		100	

Figure S2. Comparison of editing activities of BE1 and BE3 in *C. glutamicum*. Base editing efficiency and CBE-STOP efficiency of BE1 and BE3 using CBE-STOP targeting *ldh*-W134Ter. Base editing efficiency; Mutation frequency of base editing as C to T conversion, STOP efficiency; mutation frequency of base editing resulting in STOP codon

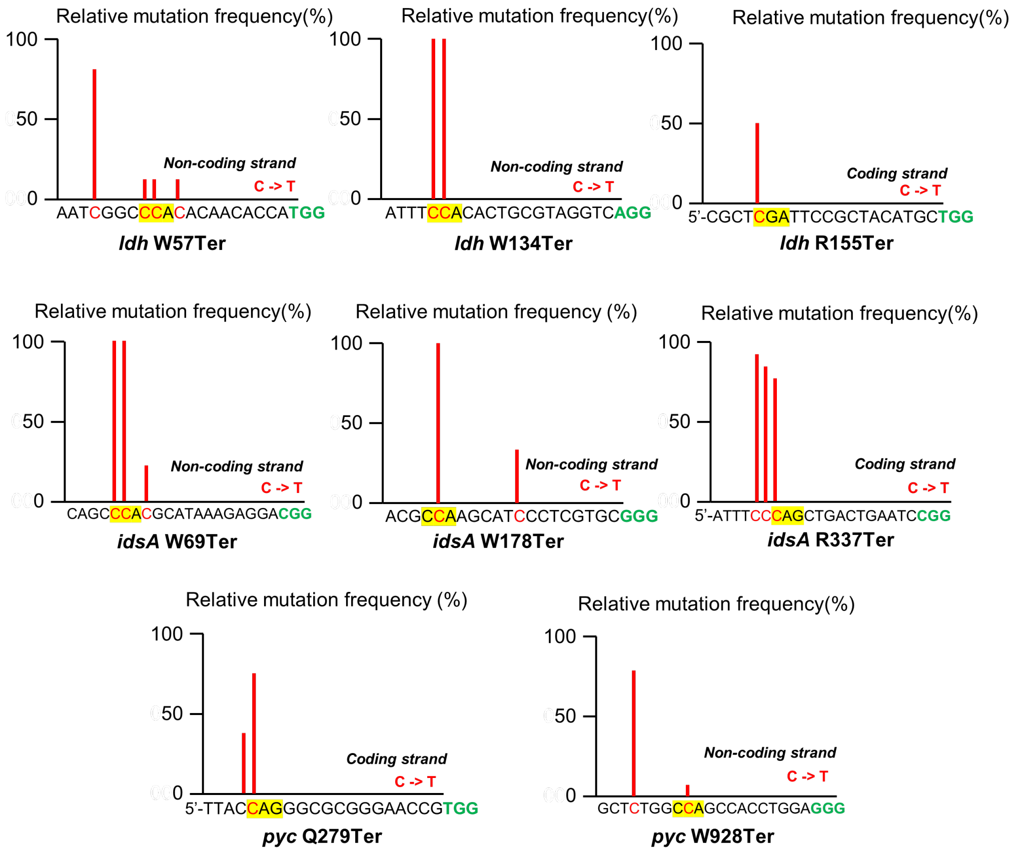

Figure S3. Nonsense mutations in different genes in *C. glutamicum* using CBE-STOP. Each target gene on the chromosome (*ldh*, *idsA,* and *pyc*) was mutated using CBE-STOP to generate STOP codons. The positions of the STOP codons are highlighted. Protospacer adjacent motif (PAM) site is shown in green. The original DNA sequences and edited DNA sequences with target codons are shown in red. STOP efficiency defines the number of any nonsense mutations confirmed among obtained strains. The relative mutation frequencies were analyzed for targeted genes.

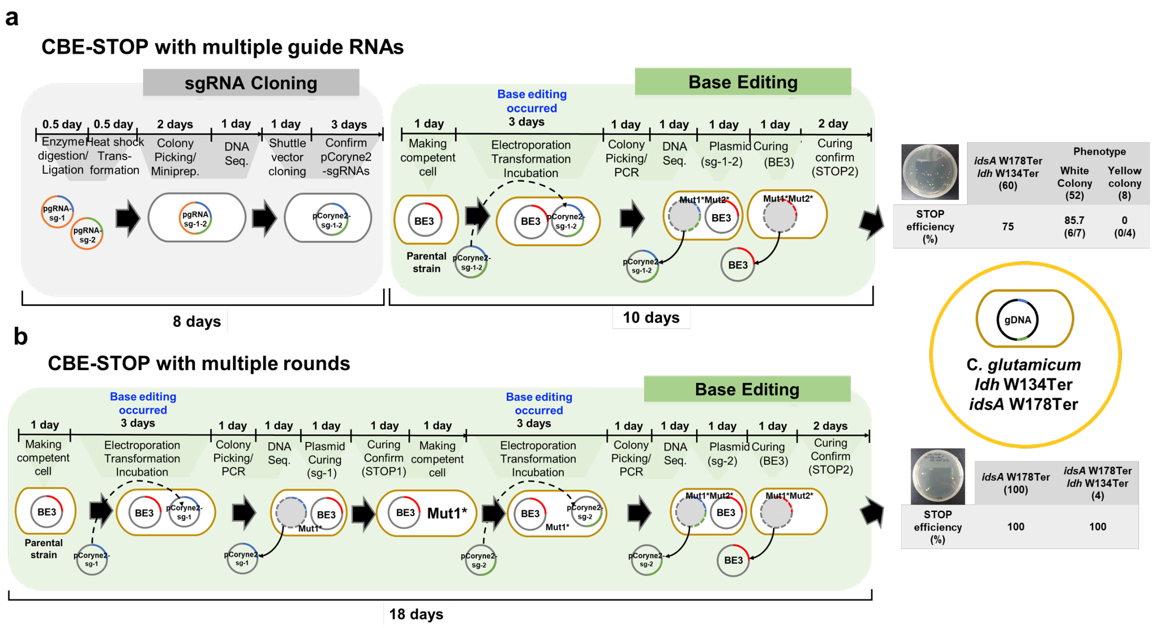

Figure S4. Comparison of the procedures for multiplexed gene editing using CBE-STOP in *C. glutamicum*. (**A**) Constructing CBE-STOP mutant strains using multiplexed sgRNA in a single editing round. pCoryne-sgRNA, including two sgRNAs targeting different genes, was used for CBE-STOP. As result, *C. glutamicum ldh*-W134Ter *idsA*-W178Ter was obtained at 75% STOP efficiency (n = 60). (**B**) Constructing CBE-STOP mutant strains through multiple editing rounds with different single sgRNAs. A repeat process of transformation and curing of pCoryne2-sgRNA was required. As result, *C. glutamicum ldh*-W134Ter *idsA*-W179Ter was obtained at 100% STOP efficiency (n = 4). Plasmids used in this work: pCoryne-sgRNA (pZ8-1 derivative, *Eco*RI, *Bgl*II, and *Bam*HI compatible, Km^{r}), pCoryne-sgRNA-idsA-ldh (pCoryne-sgRNA containing sgRNA-idsA-ldh, Km^{r}), pgRNA-idsA (pgRNA-bacteria containing sgRNA-idsA-W69Ter, Amp^{r}), pgRNA-ldh (pgRNA-bacteria containing sgRNA-ldh-W57Ter, Amp^{r}).

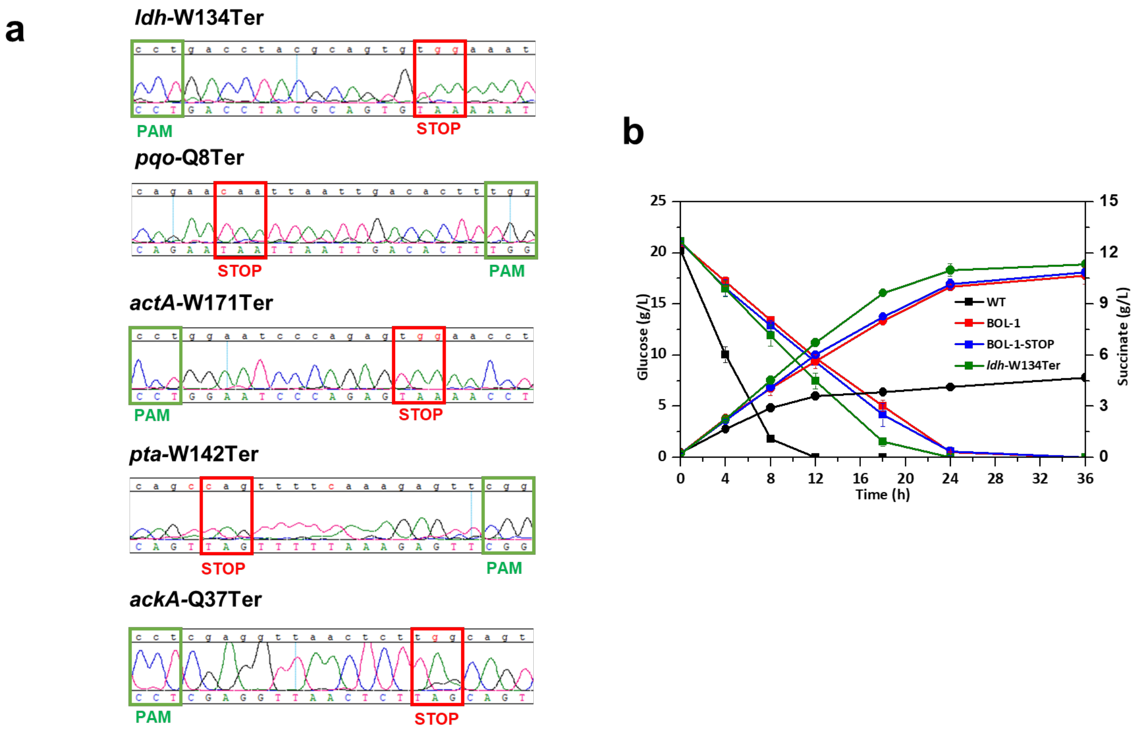

Figure S5. Sequence verification and evaluation of BOL-1-STOP strain. (**A**) The final strain, BOL-1-STOP, was confirmed by DNA Sanger sequencing. (**B**) Phenotype confirmation by flask culture. Succinate production by either BOL-1 engineered with in-frame deletions or BOL-1-STOP with CBE-STOP showed the same levels. Squares, glucose; circles, succinate. Black, WT; red, BOL-1; blue, BOL-1 STOP; green, *ldh*-W134Ter.

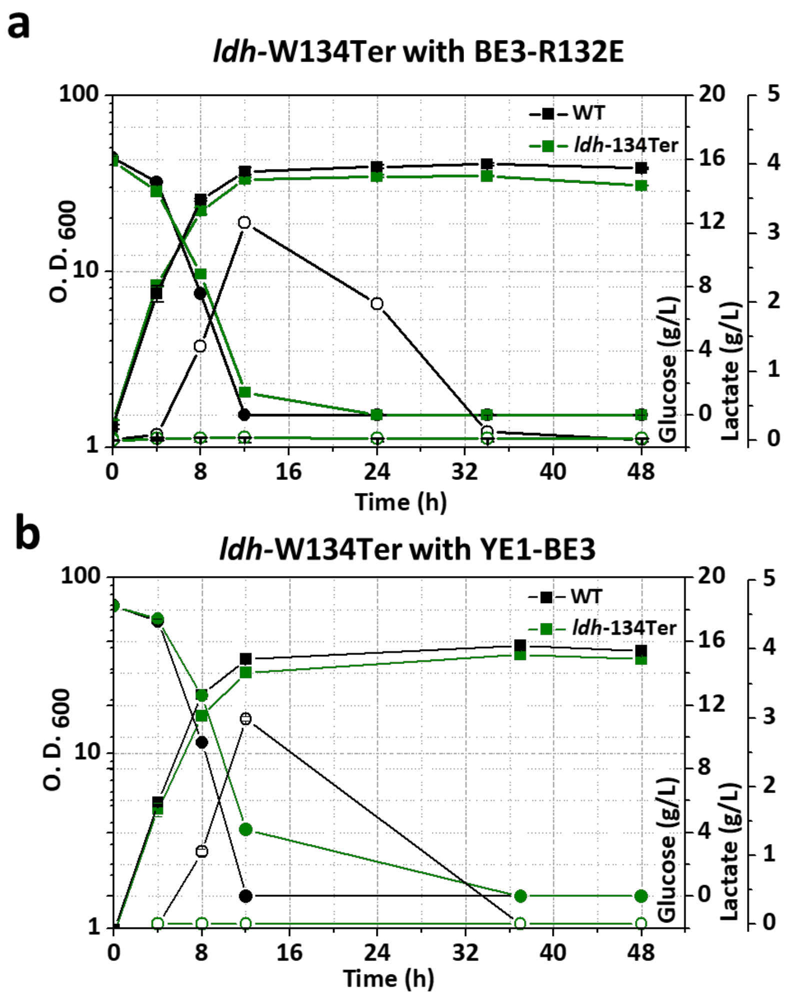

Figure S6. Cell growth and lactate production of WT and *ldh*-W134Ter strains using either BE3-R132E or YE1-BE3.

a

ackA

Name	Target protospacer
ackA-Q37Ter	GAGcagATTGGTGAGCCAAA**CGG**
ackA-Q220Ter	TGCTGTTcaaGGTGGCCGTG**CGG**
ackA-Q308Ter	CACcaaCTCCGCCGCTACCT**CGG**

	ackA-Q37Ter	ackA-Q220Ter	ackA-Q308Ter
C position for STOP	C4	C8	C4
Motif	G**C**	T**C**	C**C**
edited/ picked	0/50	0/50	0/50

b

actA

Name	Target protospacer
actA-Q22Ter	GCGGCAcagTTTGTTAACCA**CGG**
actA-Q119Ter	TCCGGCcagCAGGTTGAAGA**GGG**
actA-Q171Ter	ACTGccaAGAGTTAACCTCG**AGG**

	actA-Q22Ter	actA-Q119Ter	actA-Q171Ter	
C position for STOP	C7	C7	C5	C6
Motif	A**C**	C**C**	G**C**	C**C**
edited/ picked	0/50	0/50	0/50	0/50

Figure S7. Attempts to perform the HF-CBE-STOP for *ackA* and *actA* genes using BE3-R132E. Grey box represents a combination of C position and motif, resulting in low editing efficiency to generate the desired PTC using HF-CBE-STOP (pCoryne-BE3-R132E). Red font; target codon for PTC, bold and underline; PAM site, gray box;

Table S1. All possible BE3-STOPs using either NGG-CBE or NG-CBE.

Refer to the Supplementary Dataset 1.

Table S2. Oligonucleotides used for gene cloning.

Name	Relevant characteristics (5′ → 3′)
CBE3-fwd	AAAAAAGATCTTTTGTTTAACTTTAAGAAGG
CBE3-rev	ATATACTCGAGGAGCATCTTAATCTTGTTCT
pCoryne2-fwd	ATATAGAATTCAATCTAGACTCGAGGAGATT
pCoryne2-rev	ATATAGGATCCGCTCTCCTGAGTAGGACAAA
pCoryne2-sgRNA-fwd	taat**ACTAGT**[protospacer-20bp]GTTTTAGAGCTAGAAATAGCAA
pCoryne2-sgRNA-rev	at**ACTAGT**ATTATACCTAGGACTGAGCTAGC
pCoryne2-AarI-fwd	ata**GAATTCCACCTGC**atatGGAGcctctcctgagtagg
pCoryne2-AarI-rev	ata**GAATTC**CACCTGCatatGCCGaatctagactcgagg
sgRNA-FR1-F	atatat**CACCTGC**AATTCGGCGAATTCTAAAGATCTT
sgRNA-FR1-R	atatat**CACCTGC**AATTATGTgGATCCAGTTCACCGA
sgRNA-FR2-F	atatat**CACCTGC**AATTACATGAATTCTAAAGATCTT
sgRNA-FR2-R	atatat**CACCTGC**AATTCATGgGATCCAGTTCACCGA
sgRNA-FR3-F	atatat**CACCTGC**AATTCATGGAATTCTAAAGATCTT
sgRNA-FR3-R	atatat**CACCTGC**AATTCTCCgGATCCAGTTCACCGA

Note: Restriction enzyme sites are shown in bold. Target-specific protospacer regions of the sgRNAs are underlined.

Table S3. Target protospacer used for CBE-STOP.

Name	Relevant characteristics (5′ → 3′)	Name	Relevant characteristics (5′ → 3′)
ldh-W57Ter	AATCGGCccaCACAACACCA**TGG**	sdhA-Q294Ter	TTCATCcagTTCCACCCAAC**CGG**
ldh-W134Ter	ATTTccaCACTGCGTAGGTC**AGG**	sdhB-Q10Ter	GCGTcaaGCAGGCCCAACTG**CGG**
ldh-R155Ter	CGCTcgaTTCCGCTACATGC**TGG**	cg2425-R229Ter	GTGTCCcgaGTGGAACGAGA**CGG**
idsA-W69Ter	**CCG**TCCTCTTTATGCGtggG**CTG**	cg2452-W49Ter	**CCG**CACCGGCAACAtggACGCTT
idsA-W178Ter	**CCC**GCACGAGGGATGCTtgg**CGT**	cg2527-Q488Ter	CCTCCcagATCAACGAAAAC**TGG**
idsA-Q337Ter	ATTTCCcagCTGACTGAATC**CGG**	cg2597-W457Ter	**CCG**TCTGCACACCCTGtggGAGC
pyc-Q279Ter	TTACcagGGCGCGGGAACCG**TGG**	cg2623-Q130Ter	GGCACCcagGTCGCAGAAGG**CGG**
pyc-W928Ter	GCTCTGGCcagCCACCTGGA**GGG**	cg2651-W53Ter	**CCA**TGGGCTCACTTTGtggGACT
pta-Q96Ter	CGCACCcagGTAAACAATGC**CGG**	cg2688-W37Ter	**GGT**TTGcgaGCCGCTCTTCCTGG
pta-W142Ter	CAGccaGTTTTCAAAGAGTT**CGG**	cg2848-W201Ter	**CCC**CCGACTTCACGtggAAAATC
ackA-Q37Ter	GAGcagATTGGTGAGCCAAA**CGG**	cg3057-Q271Ter	GTCTTTcaaCGCATAACCCA**AGG**
ackA-Q220Ter	TGCTGTTcaaGGTGGCCGTG**CGG**	cg3075-W393Ter	**CCT**ACCTCATTCCAtggATGGCT
ackA-Q308Ter	CACcaaCTCCGCCGCTACCT**CGG**	cg3085-Q191Ter	GCCGAAcaaGCAGCACGCGA**TGG**
pqo-Q8Ter	AGAAcaaTTAATTGACACTT**TGG**	cg3158-Q299Ter	ACCTATcaaCTGCTCCGCAG**TGG**
actA-Q22Ter	GCGGCAcagTTTGTTAACCA**CGG**	cg3167-W185Ter	**CCA**CCATTTTGCGAtggATGAAC
actA-Q119Ter	TCCGGCcagCAGGTTGAAGA**GGG**	cg3187-W505Ter	**CCC**GACCACTTATTCGtggATCA
actA-W171Ter	ACTGccaAGAGTTAACCTCG**AGG**	cg3192-W104Ter	**CCG**AACACCCCGAGtggATCCAA
sdhCD-Q73Ter	GAAGCAcaaGTAGATGTCTA**CGG**	cg3243-W71Ter	**CCT**CGAAGGGGTGGCAtggGAAG

Note: Target bases for STOP are shown in lowercase and PAM(NGG) sites are shown in bold.

Table S5. List of the sequence-context-dependent motifs and base editing performance using CBE-STOP.

Target gene	Genotype	5'-Target protospacer with the PAM	C location	Motif (5'-NC)	# Colony	Percentage (%)
idsA	W69Ter	**CCG**TCCTCTTTATGCGtggGCTG	C4	G	0/18	0
			C5	C	18/18	100
			C6	CC	18/18	100
			C8	A	4/18	22
			C10	G	0/18	0
idsA	W178Ter	**CCC**GCACGAGGGATGCTtggCGT	C4	G	0/6	0
			C5	GC	6/6	100
			C9	G	0/6	0
			C12	T	2/6	33
idsA	Q337Ter	ATTTCCcagCTGACTGAATC**CGG**	C5	T	12/13	92
			C6	TC	11/13	84.6
			C7	CC	10/13	76.9
			C10	G	0/13	0
pyc	Q279Ter	TTACcagGGCGCGGGAACCG**TGG**	C4	A	3/8	37.5
			C5	AC	6/8	75
			C10	G	0/8	0
pyc	W928Ter	GCTCTGGCcagCCACCTGGA**GGG**	C4	T	11/14	78.6
			C8	G	0/14	0
			C9	GC	1/14	7.1
ldh	W57Ter	**CCA**TGGTGTTGTGtggGCCGATT	C4	T	13/16	81.25
			C7	G	0/16	0
			C8	GC	2/16	12.5
			C9	CC	2/16	12.5
			C11	CA	2/16	12.5
ldh	W134Ter	**CCT**GACCTACGCAGTGtggAAAT	C5	T	14/14	100
			C6	TC	14/14	100
			C8	A	0/14	0
			C10	A	0/14	0
ldh	R155Ter	CGCTcgaTTCCGCTACATGC**TGG**	C3	G	0/14	0
			C5	T	7/14	50
			C10	T	0/14	0
			C11	TC	0/14	0

Note: The lower cases in 5'-Target protospacer present the bases for CBE-STOP codons and bolded cases present NGG PAM sites.
NN represents 5' motif which have two cases dependent on base editing results. N is base that has likelihood converted to T by base editing.

Table S6. List of the off-targets for ldh-W134Ter and BOL-1-STOP samples using CBE-STOP.

Position	Mutation	Status
1994	GtoA	Both
14840	CtoT	Both
187527	CtoT	Both
255436	CtoT	Both
376382	CtoT	Both
384087	CtoT	Both
388457	GtoA	Both
433207	CtoT	Both
482935	GtoA	Both
552732	GtoA	Both
617663	GtoA	Both
675617	CtoT	Both
691874	GtoA	Both
868878	CtoT	Both
914494	CtoT	Both
945229	CtoT	Both
950040	CtoT	Both
983894	CtoT	Illumina
1189680	AtoG	Illumina
1330285	GtoA	Both
1401249	CtoT	Illumina
1421201	GtoA	Both
1498866	GtoA	Both
1602806	CtoT	Both
1632004	CtoT	Both
1659787	GtoA	Both
1694168	CtoT	Both
1737364	CtoT	Both
1780680	GtoA	Both
1796493	CtoT	Both
1816139	GtoA	Both
1821692	GtoA	Both
1823503	GtoA	Both
1851786	CtoT	Both
1851885	CtoT	Both
1892872	CtoT	Both
1904246	GtoA	Both
2011490	GtoA	Both
2037747	GtoA	Both
2083006	CtoT	Both
2299999	GtoA	Both
2346277	GtoA	Both
2396621	GtoA	Both
2484563	AtoN	Illumina
2484564	TtoN	Illumina
2484565	AtoN	Illumina
2484566	AtoN	Illumina
2484567	TtoN	Illumina
2484568	GtoN	Illumina
2484569	AtoN	Illumina
2484570	GtoN	Illumina
2484571	TtoN	Illumina
2496690	GtoA	Both
2502689	GtoA	Both
2586390	AtoC	Illumina
2586474	CtoT	Illumina
2586538	CtoT	Illumina
2586670	GtoA	Illumina
2586681	TtoC	Illumina
2586685	GtoA	Illumina
2594637	GtoA	Both
2854562	GtoA	Both
2899369	GtoA	Both
2952555	GtoA	Both
2966308	GtoA	Both
3045409	GtoA	Both
3096031	GtoA	Both
3112990	CtoT	on-target
3112991	CtoT	on-target

Note: "Both" means that it was detected by both PacBio and Illumina

Position	Pattern	Status	Position_considered_promoter	Position_info (TSS)	Position_info (TLS)	Mutation type	amino acid pattern
1994	GtoA	Both					
14840	CtoT	ldh					
187527	CtoT	Both			CDS_NCgl0173;	Silent	HtoH
255436	CtoT	Both			CDS_NCgl0235;	Silent	LtoL
376382	CtoT	Both			CDS_NCgl0347;	Silent	DtoD
384087	CtoT	ldh			CDS_NCgl0352;	Nonsense	WtoX
388457	GtoA	Both			CDS_NCgl0355;	Silent	StoS
433207	CtoT	ldh	Pro_NCgl0395;		CDS_NCgl0396;	Missense	StoF
482935	GtoA	Both	Pro_NCgl0445;				
552732	GtoA	Both			CDS_NCgl0512;	Silent	YtoY
617663	GtoA	Both			CDS_NCgl0579;	Silent	LtoL
675617	CtoT	Both			CDS_NCgl0631;	Missense	GtoE
691874	GtoA	Both	Pro_NCgl0646;Pro_NCgl0647;				
868878	CtoT	ldh	Pro_NCgl0786;				
914494	CtoT	ldh			CDS_NCgl0821;	Silent	LtoL
945229	CtoT	ldh			CDS_NCgl0856;	Silent	ItoI
950040	CtoT	Both			CDS_NCgl0858;	Missense	StoF
1330285	GtoA	Both			CDS_NCgl1216;	Missense	StoL
1421201	GtoA	ldh			CDS_rpsA;	Silent	GtoG
1498866	GtoA	Both			CDS_NCgl1366;	Silent	LtoL
1602806	CtoT	ldh			CDS_NCgl1463;	Silent	ItoI
1632004	CtoT	Both			CDS_NCgl1487;	Silent	ItoI
1659787	GtoA	Both			CDS_NCgl1509;	Silent	TtoT
1694168	CtoT	Both			CDS_NCgl1537;	Silent	VtoV
1737364	CtoT	ldh			CDS_NCgl1575;	Missense	RtoK
1780680	GtoA	Both			CDS_NCgl1613;	Silent	FtoF
1796493	CtoT	Both			CDS_NCgl1627;	Missense	StoF
1816139	GtoA	Both					
1821692	GtoA	Both			CDS_NCgl1658;	Silent	YtoY
1823503	GtoA	ldh			CDS_NCgl1659;	Silent	LtoL
1851786	CtoT	Both			CDS_NCgl1683;	Silent	LtoL
1851885	CtoT	Both			CDS_NCgl1683;	Silent	LtoL
1892872	CtoT	Both			CDS_NCgl1713;	Silent	ItoI
1904246	GtoA	Both			CDS_NCgl1721;	Silent	YtoY
2011490	GtoA	ldh		UTR_NCgl1859;			
2037747	GtoA	Both	Pro_NCgl1856;				
2083006	CtoT	Both			CDS_NCgl1900;	Silent	GtoG
2299999	GtoA	Both			CDS_NCgl2092;	Silent	EtoE
2396621	GtoA	Both			CDS_NCgl2185;	Silent	LtoL
2496690	GtoA	Both			CDS_NCgl2274;	Missense	StoF
2502689	GtoA	Both	Pro_NCgl2277;Pro_NCgl2278;				
2594637	GtoA	Both	Pro_NCgl2364;		CDS_NCgl2365;	Silent	VtoV
2854562	GtoA	Both			CDS_NCgl2590;	Silent	LtoL
2899369	GtoA	Both			CDS_NCgl2627;	Missense	StoF
2952555	GtoA	Both	Pro_NCgl2671;		CDS_NCgl2670;	Silent	PtoP
2966308	GtoA	ldh	Pro_NCgl2682;				
3045409	GtoA	Both			CDS_NCgl2757;	Silent	GtoG
3096031	GtoA	Both			CDS_NCgl2794;	Silent	LtoL
3112990	CtoT	ldh_target		UTR_NCgl2839;	CDS_ldh;	Nonsense	WtoX
3112991	CtoT	ldh_target		UTR_NCgl2839;	CDS_ldh;	Nonsense	WtoX

Note: "Pro" is considered as promoter region that is calculated by 200bp upstream of TLS

Position	Pattern	Status	Position_as_promoter	Position_info (TSS)	Position_info (TLS)	Mutation type	amino acid pattern
1994	GtoA	Both					
129668	CtoT	BOL			CDS_NCgl0115;	Silent	LtoL
139594	GtoA	BOL			CDS_NCgl0125;	Nonsense	WtoX
161743	CtoT	BOL			CDS_NCgl0148;	Silent	HtoH
169358	CtoT	BOL			CDS_NCgl0155;	Silent	FtoF
187527	CtoT	Both			CDS_NCgl0173;	Silent	HtoH
245172	CtoT	BOL			CDS_NCgl0228;	Silent	PtoP
255436	CtoT	Both			CDS_NCgl0235;	Silent	LtoL
322509	CtoT	BOL					
369715	CtoT	BOL			CDS_NCgl0341;	Silent	StoS
376382	CtoT	Both			CDS_NCgl0347;	Silent	DtoD
388457	GtoA	Both			CDS_NCgl0355;	Silent	StoS
403131	CtoT	BOL			CDS_NCgl0370;	Silent	LtoL
441019	CtoT	BOL					
480632	CtoT	BOL	Pro_NCgl0442;		CDS_NCgl0443;	Silent	GtoG
482935	GtoA	Both	Pro_NCgl0445;				
496216	CtoT	BOL					
509926	CtoT	BOL	Pro_rplJ;	UTR_NCgl0468;			
513058	CtoT	BOL			CDS_rpoB;	Missense	StoF
532435	CtoT	BOL			CDS_NCgl0485;	Missense	StoF
540317	CtoT	BOL	Pro_rplE;		CDS_rplX;	Missense	StoF
552732	GtoA	Both			CDS_NCgl0512;	Silent	YtoY
575268	CtoT	BOL					
617663	GtoA	Both			CDS_NCgl0579;	Silent	LtoL
633780	CtoT	BOL			CDS_NCgl0597;	Silent	WtoW
648122	CtoT	BOL			CDS_NCgl0607;	Missense	GtoE
675617	CtoT	Both			CDS_NCgl0631;	Missense	GtoE
691874	GtoA	Both	Pro_NCgl0646;Pro_NCgl0647;				
695529	CtoT	BOL			CDS_NCgl0650;	Silent	ItoI
728288	GtoA	BOL			CDS_NCgl0677;	Missense	StoF
733682	CtoT	BOL			CDS_NCgl0682;	Missense	StoF
736030	CtoT	BOL			CDS_NCgl0685;	Silent	FtoF
742560	GtoA	BOL	Pro_NCgl0691;	UTR_NCgl0689;	CDS_NCgl0692;	Missense	RtoQ
743993	CtoT	BOL	Pro_NCgl0694;		CDS_NCgl0695;	Silent	KtoK
762181	CtoT	BOL			CDS_NCgl0704;	Silent	LtoL
804395	CtoT	BOL	Pro_NCgl0732;Pro_NCgl0733;				
805227	GtoA	BOL			CDS_NCgl0734a;	Missense	CtoY
851264	CtoT	BOL	Pro_NCgl0776;				
854758	CtoT	BOL			CDS_NCgl0779;	Missense	StoF
895241	CtoT	BOL	Pro_NCgl0804;				
917748	GtoA	BOL	Pro_NCgl0824;				
950040	CtoT	Both			CDS_NCgl0858;	Missense	StoF
1043134	GtoA	BOL					
1085913	CtoT	BOL			CDS_NCgl0989;	Silent	ItoI
1217421	GtoA	BOL	Pro_NCgl1114;				
1278686	CtoT	BOL			CDS_NCgl1165;	Silent	FtoF
1311026	CtoT	BOL			CDS_NCgl1197;	Silent	GtoG
1330285	GtoA	Both			CDS_NCgl1216;	Missense	StoL
1337832	GtoA	BOL	Pro_NCgl1221;				
1340996	CtoT	BOL			CDS_NCgl1224;	Silent	FtoF
1348279	CtoT	BOL					
1406118	CtoT	BOL			CDS_NCgl1289;	Silent	LtoL
1434851	CtoT	BOL			CDS_NCgl1315;	Silent	ItoI
1479049	CtoT	BOL	Pro_NCgl1354a;				
1498866	GtoA	Both			CDS_NCgl1366;	Silent	LtoL
1542995	CtoT	BOL	Pro_NCgl1408;				
1563254	GtoA	BOL			CDS_NCgl1428;	Silent	LtoL
1565926	GtoA	BOL			CDS_NCgl1430;	Silent	VtoV
1605514	CtoT	BOL			CDS_NCgl1466;	Silent	DtoD
1624522	GtoA	BOL			CDS_NCgl1480;	Missense	StoF
1626758	CtoT	BOL			CDS_NCgl1482;	Silent	FtoF
1632004	CtoT	Both			CDS_NCgl1487;	Silent	ItoI
1637190	GtoA	BOL			CDS_NCgl1490;	Missense	RtoK
1659787	GtoA	Both			CDS_NCgl1509;	Silent	TtoT
1694168	CtoT	Both			CDS_NCgl1537;	Silent	VtoV
1722127	CtoT	BOL			CDS_NCgl1564;	Silent	LtoL
1730270	CtoT	BOL			CDS_NCgl1571;	Silent	PtoP
1762480	CtoT	BOL			CDS_ruvB;	Silent	FtoF
1776652	GtoA	BOL		UTR_NCgl1610;			
1780680	GtoA	Both			CDS_NCgl1613;	Silent	FtoF
1796493	CtoT	Both			CDS_NCgl1627;	Missense	StoF
1816139	GtoA	Both					
1821692	GtoA	Both			CDS_NCgl1658;	Silent	YtoY
1849841	GtoA	BOL			CDS_NCgl1681;	Silent	NtoN
1851786	CtoT	Both			CDS_NCgl1683;	Silent	LtoL
1851885	CtoT	Both			CDS_NCgl1683;	Silent	LtoL
1856384	CtoT	BOL			CDS_NCgl1687;	Silent	StoS
1862484	CtoT	BOL					
1867241	CtoT	BOL			CDS_NCgl1697;	Silent	FtoF
1874379	GtoA	BOL			CDS_NCgl1702;	Silent	FtoF
1892872	CtoT	Both			CDS_NCgl1713;	Silent	ItoI
1904246	GtoA	Both			CDS_NCgl1721;	Silent	YtoY
1990241	CtoT	BOL			CDS_NCgl1812;	Missense	StoN
1990279	TtoA	BOL			CDS_NCgl1812;	Missense	QtoH
1990285	TtoC	BOL			CDS_NCgl1812;	Silent	StoS
1990296	CtoT	BOL			CDS_NCgl1812;	Silent	FtoF
1990303	TtoA	BOL			CDS_NCgl1812;	Silent	AtoA
1990309	AtoC	BOL			CDS_NCgl1812;	Silent	StoS

1990318	AtoG	BOL			CDS_NCgl1812;	Silent	CtoC
1990321	AtoT	BOL			CDS_NCgl1812;	Missense	DtoE
1990331	TtoC	BOL			CDS_NCgl1812;	Missense	NtoS
1990347	TtoC	BOL			CDS_NCgl1812;	Silent	LtoL
1990393	CtoT	BOL			CDS_NCgl1812;	Silent	KtoK
1990691	AtoT	BOL			CDS_NCgl1812;	Missense	VtoD
1992946	CtoG	BOL			CDS_NCgl1815;	Silent	VtoV
1993681	GtoA	BOL			CDS_NCgl1815;	Silent	YtoY
1993797	GtoT	BOL			CDS_NCgl1815;	Silent	LtoL
1993912	CtoT	BOL			CDS_NCgl1815;	Silent	PtoP
1993948	GtoC	BOL			CDS_NCgl1815;	Silent	RtoR
1993954	GtoA	BOL			CDS_NCgl1815;	Silent	TtoT
1993956	TtoC	BOL			CDS_NCgl1815;	Silent	DtoD
1993966	TtoC	BOL			CDS_NCgl1815;	Silent	RtoR
1993984	GtoA	BOL			CDS_NCgl1815;	Silent	GtoG
1993987	CtoT	BOL			CDS_NCgl1815;	Silent	TtoT
1994000	AtoG	BOL			CDS_NCgl1815;	Missense	VtoA
1994011	GtoA	BOL			CDS_NCgl1815;	Silent	TtoT
1994014	GtoT	BOL			CDS_NCgl1815;	Silent	TtoT
1994016	TtoC	BOL			CDS_NCgl1815;	Silent	AtoA
1994017	TtoA	BOL			CDS_NCgl1815;	Silent	AtoA
1994025	GtoA	BOL			CDS_NCgl1815;	Silent	RtoR
1994032	AtoG	BOL			CDS_NCgl1815;	Silent	GtoG
1994038	GtoA	BOL			CDS_NCgl1815;	Silent	NtoN
1994059	TtoC	BOL			CDS_NCgl1815;	Silent	EtoE
1994062	TtoC	BOL			CDS_NCgl1815;	Silent	LtoL
1994064	GtoC	BOL			CDS_NCgl1815;	Silent	QtoQ
1994068	GtoA	BOL			CDS_NCgl1815;	Silent	DtoD
1994086	TtoC	BOL			CDS_NCgl1815;	Silent	VtoV
1994107	AtoG	BOL			CDS_NCgl1815;	Silent	VtoV
1994116	AtoG	BOL			CDS_NCgl1815;	Silent	RtoR
1994122	GtoT	BOL	Pro_NCgl1815;				
1994134	TtoG	BOL	Pro_NCgl1815;				
1994149	CtoT	BOL	Pro_NCgl1815;				
1994155	GtoA	BOL	Pro_NCgl1815;				
1994164	GtoA	BOL	Pro_NCgl1815;				
1994188	TtoG	BOL	Pro_NCgl1815;				
1994205	CtoT	BOL	Pro_NCgl1815;				
1994226	TtoA	BOL	Pro_NCgl1815;				
1994231	TtoA	BOL	Pro_NCgl1815;				
1994250	CtoT	BOL	Pro_NCgl1815;				
1994254	TtoA	BOL	Pro_NCgl1815;				
1994257	TtoC	BOL	Pro_NCgl1815;				
1994281	AtoG	BOL	Pro_NCgl1815;				
1994290	GtoA	BOL	Pro_NCgl1815;				
1994304	AtoG	BOL	Pro_NCgl1815;				
1994312	GtoC	BOL	Pro_NCgl1815;				
1994322	CtoT	BOL					
1994347	CtoT	BOL					
1994359	CtoT	BOL					
2020826	CtoT	BOL	Pro_NCgl1844;				
2037747	GtoA	Both	Pro_NCgl1856;				
2083006	CtoT	Both			CDS_NCgl1900;	Silent	GtoG
2104260	GtoA	BOL			CDS_NCgl1918;	Silent	QtoQ
2118788	CtoT	BOL			CDS_NCgl1929;	Missense	StoF
2135728	GtoA	BOL	Pro_NCgl1944;Pro_NCgl1945;				
2160839	CtoT	BOL					
2216756	CtoT	BOL			CDS_NCgl2020;	Missense	GtoE
2230702	GtoA	BOL			CDS_NCgl2032;	Missense	StoF
2252815	GtoA	BOL			CDS_NCgl2050;	Nonsense	WtoX
2299999	GtoA	Both			CDS_NCgl2092;	Silent	EtoE
2376942	GtoA	BOL					
2378813	GtoA	BOL					
2379389	GtoA	BOL	Pro_NCgl2165;Pro_NCgl2166;				
2396621	GtoA	Both			CDS_NCgl2185;	Silent	LtoL
2429386	GtoA	BOL			CDS_NCgl2216;	Missense	StoF
2484562	GtoA	BOL	Pro_NCgl2259;Pro_NCgl2260;				
2484574	GtoA	BOL	Pro_NCgl2259;Pro_NCgl2260;				
2496690	GtoA	Both			CDS_NCgl2274;	Missense	StoF
2502689	GtoA	Both	Pro_NCgl2277;Pro_NCgl2278;				
2539521	GtoA	BOL			CDS_NCgl2313;	Missense	StoF
2594637	GtoA	Both	Pro_NCgl2364;		CDS_NCgl2365;	Silent	VtoV
2622611	CtoT	BOL					
2654667	CtoT	BOL	Pro_NCgl2418;				
2670898	GtoA	BOL	Pro_NCgl2436;				
2672441	GtoA	BOL			CDS_NCgl2437;	Silent	FtoF
2672846	CtoT	BOL	Pro_NCgl2437;				
2718166	GtoA	BOL					
2720170	GtoA	BOL			CDS_NCgl2471;	Silent	EtoE
2730372	CtoT	actA_target			CDS_NCgl2480;	Nonsense	WtoX
2730373	CtoT	actA_target			CDS_NCgl2480;	Nonsense	WtoX
2741856	GtoA	BOL			CDS_NCgl2491;	Silent	LtoL
2778484	GtoA	pqo_target			CDS_NCgl2521;	Nonsense	QtoX
2832035	GtoA	BOL	Pro_NCgl2574;				
2854562	GtoA	Both			CDS_NCgl2590;	Silent	LtoL
2894225	CtoT	BOL			CDS_NCgl2625;	Silent	TtoT
2899369	GtoA	Both			CDS_NCgl2627;	Missense	StoF
2936398	GtoA	ackA_target			CDS_NCgl2656;	Nonsense	EtoX
2937469	CtoT	pta_target			CDS_eutD;	Nonsense	WtoX
2937470	CtoT	pta_target			CDS_eutD;	Nonsense	WtoX
2937477	CtoT	BOL			CDS_eutD;	Silent	FtoF
2952555	GtoA	Both	Pro_NCgl2671;		CDS_NCgl2670;	Silent	PtoP
2997798	GtoA	BOL	Pro_NCgl2711;		CDS_NCgl2712;	Missense	GtoE
3009841	GtoA	BOL			CDS_NCgl2724;	Missense	StoF
3027963	GtoA	BOL			CDS_NCgl2741;	Silent	VtoV
3045409	GtoA	Both			CDS_NCgl2757;	Silent	GtoG
3096031	GtoA	Both			CDS_NCgl2794;	Silent	LtoL
3109053	GtoA	BOL			CDS_NCgl2808;	Silent	LtoL
3112990	CtoT	ldh_target		UTR_NCgl2839;	CDS_ldh;	Nonsense	WtoX
3112991	CtoT	ldh_target		UTR_NCgl2839;	CDS_ldh;	Nonsense	WtoX
3118643	CtoT	BOL			CDS_NCgl2816;	Missense	AtoV
3198583	GtoA	BOL			CDS_NCgl2895;	Silent	FtoF
3303441	GtoA	BOL			CDS_NCgl2987;	Silent	FtoF
3308530	GtoA	BOL	Pro_rnpA;		CDS_rpmH;	Silent	FtoF

Note: "Pro" is considered as promoter region that is calculated by 200bp upstream of TLS

Table S7. List of off-targets for *ldh*-W134Ter using HF-CBE-STOP.

YE1-BE3	
**Position**	Mutation
**569490**	G to A
**983894**	C to T
**1025242**	G to A
**2586390**	A to C
**2586474**	C to T
**2586538**	C to T
**2743517**	G to A
**3093399**	T to C

BE3-R132E	
**Position**	Mutation
**2556658**	C to G
**2610382**	C to T
**2743517**	G to A

Table S9. List of the sequence-context-dependent motifs and base editing performance using HF-CBE-STOP.

Target gene	Genotype	5'-Target protospace with the PAM	C location	Motif (5'-NC)	# Colony	Percentage(%)	# Colony	Percentage(%)
cg2425	R229Ter	GTGTCCcgaGTGGAACGAGA**CGG**	C5	T	6	100	5	100
			C6	TC	6	100	5	100
			C7	TCC	6	100	2	40
cg2452	W49Ter	**CCG**CACCGGCAACAtggACGCTT	C4	G	0	0	0	0
			C7	T	5	83.3	0	0
			C8	C	5	83.3	0	0
cg2527	Q488Ter	CCTCCcagATCAACGAAAAC**TGG**	C4	T	5	83.3	3	60
			C5	TC	5	83.3	5	100
			C6	TCC	5	83.3	5	100
			C11	T	0	0	0	0
cg2597	W457Ter	**CCG**TCTGCACACCCTGtggGAGC	C4	T	6	100	0	0
			C5	TC	6	100	0	0
			C6	TCC	6	100	0	0
			C8	A	0	0	0	0
cg2623	Q130Ter	GGCACCcagGTCGCAGAAGG**CGG**	C3	G	0	0	0	0
			C5	A	6	100	5	83.3
			C6	AC	6	100	5	83.3
			C7	ACC	6	100	5	83.3
cg2651	W53Ter	**CCA**TGGGCTCACTTTGtggGACT	C4	T	3	50	6	100
			C5	TC	3	50	6	100
			C6	TCC	3	50	6	100
			C8	A	0	0	0	0
cg2688	W37Ter	GGTTTGcgaGCCGCTCTTCCTGG	C7	G	0	0	0	0
			C11	G	0	0	0	0
cg2848	W201Ter	**CCC**CCGACTTCACGtggAAAATC	C7	T	6	100	0	0
			C8	TC	2	33.3	0	0
			C10	A	0	0	0	0
cg3057	Q271Ter	GTCTTTcaaCGCATAACCCA**AGG**	C3	T	6	100	0	0
			C7	T	6	100	0	0
			C10	A	0	0	0	0
cg3075	W393Ter	**CCT**ACCTCATTCCAtggATGGCT	C3	G	0	0	0	0
			C4	GC	0	0	0	0
			C7	T	3	50	0	0
			C8	TC	3	50	0	0
cg3085	Q191Ter	GCCGAAcaaGCAGCACGCGA**TGG**	C3	C	0	0	0	0
			C7	A	4	80	0	0
			C11	G	0	0	0	0
cg3158	Q299Ter	ACCTATcaaCTGCTCCGCAG**TGG**	C3	C	0	0	0	0
			C7	T	6	100	0	0
			C10	A	0	0	0	0
cg3167	W185Ter	**CCA**CCATTTTGCGAtggATGAAC	C4	T	2	33.3	0	0
			C7	T	6	100	0	0
			C8	TC	2	33.3	0	0
			C11	T	1	16.7	0	0
cg3187	W505Ter	**CCC**GACCACTTATTCGtggATCA	C5	T	6	100	6	100
			C6	TC	6	100	6	100
			C8	A	6	100	6	100
cg3192	W104Ter	**CCG**AACACCCCGAGtggATCCAA	C7	T	5	83.3	0	0
			C8	TC	4	66.7	0	0
			C10	A	0	0	0	0
cg3243	W71Ter	**CCT**CGAAGGGGTGGCAtggGAAG	C4	T	6	100	6	100
			C5	TC	6	100	6	100
			C6	TCC	6	100	6	100
			C10	G	0	0	0	0
			C11	GC	0	0	0	0

Note: The lower cases in 5'-Target protospacer present the bases for CBE-STOP codons and bolded cases present NGG PAM sites.
NN represents 5'-motif which have two cases dependent on base editing results. N is base that has likelihood converted to T by base editing.

Table S11. List of the off-targets identified through MGEs using pCoryne-BE3-R132E.

ldh-pqo-pta						
Position	Pattern	Position_considered_promoter	Position_info (TSS)	Position_info (TLS)	Mutation type	amino acid pattern
414862	CtoT			CDS_NCgl0380;	Missense	StoL
444730	CtoT			CDS_NCgl0407;	Silent	ItoI
576192	CtoT		UTR_NCgl0541;	CDS_NCgl0540;	Silent	ItoI
686080	CtoT			CDS_NCgl0640;	Silent	FtoF
754335	CtoT			CDS_NCgl0701;	Silent	YtoY
905382	GtoA	Pro_NCgl0814;		CDS_NCgl0813;	Silent	StoS
984225	CtoA			CDS_NCgl0891;	Silent	NtoN
984305	TtoC			CDS_NCgl0891;	Silent	DtoD
984306	CtoA			CDS_NCgl0891;	Silent	DtoD
1047201	GtoA			CDS_NCgl0950;	Silent	TtoT
1093055	GtoA	Pro_NCgl0995;		CDS_NCgl0994;	Silent	EtoE
1411906	GtoA			CDS_NCgl1296;	Missense	StoF
1423575	CtoT			CDS_NCgl1305;	Missense	StoF
1508169	CtoT			CDS_NCgl1376;	Nonsense	WtoX
1518676	GtoA			CDS_NCgl1384;	Silent	DtoD
1603093	GtoA			CDS_NCgl1463;	Missense	GtoE
1628815	GtoA			CDS_NCgl1482;	Missense	RtoH
1774833	CtoT			CDS_NCgl1610;	Missense	GtoE
1995680	CtoT					
2101238	GtoA			CDS_NCgl1915;	Silent	AtoA
2488904	GtoA			CDS_NCgl2265;	Missense	StoF
2581528	GtoA			CDS_NCgl2353;	Silent	ItoI
2610150	GtoA			CDS_NCgl2377;	Missense	StoF
2743517	GtoA			CDS_NCgl2492;	Silent	RtoR
2989968	GtoA	Pro_NCgl2705;		CDS_NCgl2706;	Missense	StoF
3055940	GtoA		UTR_NCgl2783;	CDS_trmB;	Silent	FtoF

Note: "Pro" is considered as promoter region that is calculated by 200bp upstream of TLS

sdhCD-sdhA-sdhB						
Position	Pattern	Position_as_promoter	Position_info (TSS)	Position_info (TLS)	Mutation type	amino acid pattern
175523	GtoA			CDS_NCgl0160;	Silent	TtoT
374155	CtoT			CDS_NCgl0345;	Missense	TtoI
667963	CtoT	Pro_NCgl0625;				
1008515	CtoT	Pro_NCgl0914;				
1028139	CtoT			CDS_NCgl0927;	Silent	LtoL
1147611	CtoT			CDS_NCgl1055;	Missense	StoF
1256506	GtoA			CDS_NCgl1145;	Silent	AtoA
1390811	GtoA			CDS_NCgl1272;	Silent	HtoH
1447882	GtoA			CDS_infC;	Silent	PtoP
1690171	CtoT			CDS_ribH;	Silent	LtoL
1774651	CtoT			CDS_NCgl1610;	Silent	TtoT
1775128	CtoT	Pro_NCgl1610;				
1928469	CtoT	Pro_NCgl1741;				
1986063	GtoT			CDS_NCgl1806;	Silent	VtoV
2306269	CtoT	Pro_NCgl2097;				
2414198	GtoC			CDS_NCgl2201;	Missense	QtoH
2463295	CtoT	Pro_NCgl2242;		CDS_NCgl2243;	Silent	LtoL
2493641	CtoT			CDS_NCgl2271;	Silent	FtoF
2614964	CtoT			CDS_NCgl2382;	Silent	FtoF
2934649	GtoA			CDS_NCgl2655;	Missense	RtoQ
2956849	GtoA			CDS_NCgl2675;	Silent	AtoA
3190869	CtoT			CDS_NCgl2887;	Missense	StoF
3308726	GtoA	Pro_rpmH;				

sdhCD-sdhA-sdhB-pta						
Position	Pattern	Position_as_promoter	Position_info (TSS)	Position_info (TLS)	Mutation type	amino acid pattern
175523	GtoA			CDS_NCgl0160;	Silent	TtoT
178240	CtoT	Pro_NCgl0163;				
374155	CtoT			CDS_NCgl0345;	Missense	TtoI
1008515	CtoT	Pro_NCgl0914;				
1147611	CtoT			CDS_NCgl1055;	Missense	StoF
1256506	GtoA			CDS_NCgl1145;	Silent	AtoA
1390811	GtoA			CDS_NCgl1272;	Silent	HtoH
1441236	GtoA			CDS_NCgl1320;	Silent	LtoL
1661754	GtoA			CDS_NCgl1510;	Silent	PtoP
1690171	CtoT			CDS_ribH;	Silent	LtoL
1774651	CtoT			CDS_NCgl1610;	Silent	TtoT
1775128	CtoT	Pro_NCgl1610;				
1928469	CtoT	Pro_NCgl1741;				
1986063	GtoT			CDS_NCgl1806;	Silent	VtoV
2306269	CtoT	Pro_NCgl2097;				
2463295	CtoT	Pro_NCgl2242;		CDS_NCgl2243;	Silent	LtoL
2493641	CtoT			CDS_NCgl2271;	Silent	FtoF
2527345	GtoA			CDS_NCgl2302;	Silent	AtoA
2586390	AtoC			CDS_NCgl2356;	Silent	TtoT
2586474	CtoT			CDS_NCgl2356;	Silent	StoS
2586538	CtoT			CDS_NCgl2356;	Silent	NtoN
2587008	CtoT			CDS_NCgl2356;	Silent	RtoR
2587029	CtoA			CDS_NCgl2356;	Silent	PtoP
2587071	CtoT			CDS_NCgl2356;	Silent	DtoD
2614964	CtoT			CDS_NCgl2382;	Silent	FtoF
2678823	GtoA			CDS_NCgl2443;	Silent	ItoI
2956849	GtoA			CDS_NCgl2675;	Silent	AtoA
3190869	CtoT			CDS_NCgl2887;	Missense	StoF
3308726	GtoA	Pro_rpmH;				
Note: "Pro" is considered as promoter region that is calculated by 200bp upstream of TLS						
